# Burden of malaria in pregnancy among adolescent girls compared to adult women in 5 sub-Saharan African countries: A secondary individual participant data meta-analysis of 2 clinical trials

**DOI:** 10.1371/journal.pmed.1004084

**Published:** 2022-09-02

**Authors:** Clara Pons-Duran, Ghyslain Mombo-Ngoma, Eusebio Macete, Meghna Desai, Mwaka A. Kakolwa, Rella Zoleko-Manego, Smaïla Ouédragou, Valérie Briand, Anifa Valá, Abdunoor M. Kabanywanyi, Peter Ouma, Achille Massougbodji, Esperança Sevene, Michel Cot, John J. Aponte, Alfredo Mayor, Laurence Slutsker, Michael Ramharter, Clara Menéndez, Raquel González

**Affiliations:** 1 ISGlobal, Hospital Clínic-Universitat de Barcelona, Barcelona, Spain; 2 Consorcio de Investigación Biomédica en Red de Epidemiología y Salud Pública (CIBERESP), Madrid, Spain; 3 Centre de Recherches Médicales de Lambaréné (CERMEL), Lambaréné, Gabon; 4 Institute of Tropical Medicine, Travel Medicine and Human Parasitology, University Clinics, Eberhard Karls University Tübingen, Tübingen, Germany; 5 Department of Tropical Medicine, Bernhard Nocht Institute for Tropical Medicine & Dept. of Medicine University Medical Center Hamburg-Eppendorf, Hamburg, Germany; 6 Manhiça Health Research Center (CISM), Manhiça, Mozambique; 7 Malaria Branch, Division of Parasitic Diseases and Malaria, Center for Global Health, Centers for Disease Control and Prevention, Atlanta, Georgia, United States of America; 8 Ifakara Health Institute, Dodoma, Tanzania; 9 Département de santé publique, Unité de formation en sciences de la santé, Université Joseph Ki-Zerbo, Ouagadougou, Burkina Faso; 10 Faculté de Sciences de la Santé, Université d’Abomey-Calavi, Cotonou, Bénin; 11 Université de Paris, MERIT, IRD, Paris, France; 12 IRD, Inserm, Université de Bordeaux, IDLIC team, UMR 1219, Bordeaux, France; 13 Department of Medical Laboratory Sciences, Maseno University School of Medicine, Kenya; 14 Department of Physiological Science, Clinical Pharmacology, Faculty of Medicine, Eduardo Mondlane University, Maputo, Mozambique; 15 PATH, Malaria and NTDs, Seattle, Washington, United States of America; Mahidol-Oxford Tropical Medicine Research Unit, THAILAND

## Abstract

**Background:**

Malaria is among the top causes of death in adolescent girls (10 to 19 years) globally. Adolescent motherhood is associated with increased risk of adverse maternal and neonatal outcomes. The interaction of malaria, adolescence, and pregnancy is especially relevant in malaria endemic areas, where rates of adolescent pregnancy are high. However, data on burden of malaria among adolescent girls are limited. This study aimed at investigating whether adolescent girls were at a greater risk of experiencing malaria-related outcomes in pregnancy—parasitaemia and clinical disease—than adult women.

**Methods and findings:**

An individual secondary participant-level meta-analysis was conducted using data from 5,804 pregnant women participating in 2 malaria prevention clinical trials in Benin, Gabon, Kenya, Mozambique, and Tanzania between 2009 and 2014. Of the sample, 1,201 participants were adolescent girls with a mean age of 17.5 years (standard deviation (SD) 1.3) and 886 (73.8%) of them primigravidae. Among the 4,603 adult women with mean age of 27.0 years (SD 5.4), 595 (12.9%) were primigravidae. Mean gestational age at enrolment was 20.2 weeks (SD 5.2) and 1,069 (18.4%) participants were HIV-infected. Women were followed monthly until the postpartum visit (1 month to 6 weeks after delivery). This study considered outcomes including clinical episodes during pregnancy, peripheral parasitaemia at delivery, and placental malaria. A 2-stage meta-analysis approach was followed by pooling single multivariable regression results into standard DerSimonian–Laird random-effects models. Adolescent girls were more likely than adult women to present with clinical malaria during pregnancy (incidence risk ratio (IRR) 1.70, 95% confidence interval (CI) 1.20; 2.39, *p*-value = 0.003, I^2^ = 0.0%, *N* = 4,092), peripheral parasitaemia at delivery (odds ratio (OR) 2.28, 95% CI 1.46; 3.55, *p*-value < 0.001, I^2^ = 0.0%, *N* = 3,977), and placental infection (OR 1.97, 95% CI 1.31; 2.98, *p*-value = 0.001, I^2^ = 1.4%, *N* = 4,797). Similar associations were observed among the subgroup of HIV-uninfected participants: IRR 1.72 (95% CI 1.22; 2.45, *p*-value = 0.002, I^2^ = 0.0%, *N* = 3,531) for clinical malaria episodes, OR 2.39 (95% CI 1.49; 3.86, *p*-value < 0.001, I^2^ = 0.0%, *N* = 3,053) for peripheral parasitaemia, and OR 1.88 (95% CI 1.06 to 3.33, *p*-value = 0.03, I^2^ = 34.9%, *N* = 3,847) for placental malaria. Among HIV-infected subgroups statistically significant associations were not observed. Similar associations were found in the subgroup analysis by gravidity. The small sample size and outcome prevalence in specific countries limited the inclusion of some countries in the meta-analysis. Furthermore, peripheral parasitaemia and placental malaria presented a considerable level of missing data—12.6% and 18.2% of participants had missing data on those outcomes, respectively. Given the original scope of the clinical trials, asymptomatic malaria infection was only assessed at the end of pregnancy through peripheral and placental parasitaemia.

**Conclusions:**

In this study, we observed that adolescent girls in sub-Saharan Africa (SSA) are more prone to experience clinical malaria episodes during pregnancy and have peripheral malaria and placental infection at delivery than adult women. Moreover, to the best of our knowledge, for the first time this study disaggregates figures and stratifies analyses by HIV infection. Similar associations were found for both HIV-infected and uninfected women, although those for HIV-infected participants were not statistically significant. Our finding suggests that adolescent girls may benefit from targeted malaria prevention strategies even before they become pregnant.

## Abstract 2

In a secondary analysis of clinical trial data, Clara Pons-Duran and colleagues study clinical malaria during pregnancy among adolescent and adult women in Benin, Gabon, Kenya, Mozambique, and Tanzania between 2009 and 2014.

## Author summary

**Why was this study done?:**

- Both malaria infection and adolescence are associated with increased risk of adverse pregnancy and birth outcomes.
- Few studies conducted in sub-Saharan Africa (SSA) have assessed the burden of malaria in pregnancy among adolescent girls compared to adult women.
- However, there is still a need to determine the magnitude of the association between a wide range of malaria in pregnancy indicators and adolescence accounting for the number of previous pregnancies and considering special population subgroups such as HIV-infected women.

**What did the researchers do and find?:**

- We designed a secondary analysis of data of 2 clinical trials conducted between 2009 and 2014 in 5 SSA countries: Benin, Gabon, Mozambique, Tanzania, and Kenya.
- We followed an individual participant data meta-analysis approach using data from 5,804 trial participants, of whom 1,202 were adolescent girls, and including HIV-infected individuals (18.4% of the total sample).
- We observed that adolescent girls presented with clinical malaria during pregnancy more frequently than adult women (1.7 times), and that they were more likely to have experienced peripheral and placental malaria infection at delivery compared to adults (odds ratio (OR) 2.28 and 1.97, respectively).

**What do these findings mean?:**

- These findings indicate that there is an urgent need for more malaria in pregnancy research focused on adolescent girls.
- Adolescent girls may benefit from targeted malaria control strategies, even before they become pregnant, that consider their specific needs.
- To interpret our results, some limitations should be considered, namely the small sample size in specific study countries that limited their inclusion in some meta-analyses and the considerable level of missing data.

## Introduction

Malaria infection during pregnancy constitutes a major public health problem, especially in sub-Saharan Africa (SSA) where an estimated 11.6 million pregnancies were exposed to the infection in 2020 [1]. Malaria in pregnancy increases the risk of maternal morbidity and mortality, adverse pregnancy outcomes, low birth weight, and infant mortality [2,3]. Malaria infection is also one of the top causes of death among adolescent girls (10 to 19 years) globally [4].

Adolescent motherhood is associated with increased risk of preterm birth, low birth weight, and childbirth complications such as asphyxia or neonatal mortality [5], both as result of mother’s younger age and the fact that these are typically first pregnancies, a factor that is independently related to severe adverse pregnancy outcomes [6]. Studies carried out in SSA in the late 1900s showed that malaria was among the most common causes of maternal mortality in pregnant adolescents in endemic areas [7,8].

The interaction of malaria, adolescence, and pregnancy is especially relevant in SSA, where the highest malaria burden overlaps with a high rate of adolescent pregnancy. Among older adolescents (aged 15 to 19 years), it is estimated that approximately 9 million become pregnant in Africa every year [9]. Of note, the fertility rate among adolescents from SSA was deemed to be 100 births per 1,000 girls in 2019 [10]. However, literature about malaria characteristics among this population group is limited.

Recent studies have evaluated and described the burden of malaria among adolescent girls and nulliparas [11]; however, there is lack of studies assessing the adjusted association between age group and different malaria in pregnancy indicators. We identified studies performed in Tanzania, Gabon, Cameroon, and Malawi reporting on prevalence of malaria parasitaemia at delivery, and one from Papua New Guinea on placental malaria, among adolescent girls compared to adult women [12–16]. Only 3 of the studies adjusted or stratified the analyses for several confounders including gravidity and parity [13–15]. Additional identified studies reported associations between adolescence or young age with malaria infection at first antenatal care clinic visit [11,17–23].

To our knowledge, no multicountry studies to date have been done to investigate the burden of malaria in pregnancy among adolescent girls compared to adult women. Furthermore, the authors could not identify any studies that stratified results by human immunodeficiency virus (HIV) status of women. Although malaria infections among adolescent girls are known to be generally asymptomatic and incidence of clinical disease is low among them [24,25], pregnant women are known to be more likely to report signs and symptoms of malaria than non-pregnant women [26]. In this regard, we found studies evaluating incidence of symptomatic malaria among non-pregnant adolescents, but no studies that considered incidence of clinical malaria episodes during pregnancy.

In this context, we designed a secondary analysis of 2 multicountry clinical trials to address the abovementioned gaps. It was hypothesised that adolescence compared to adulthood is significantly associated with increased burden of malaria in pregnancy.

## Methods

### Study design

This is an individual participant-level data meta-analysis of 2 clinical trials. The selection of the included studies was not systematic; only the data collected as part of the Malaria in Pregnancy Preventive Alternative Drugs (MiPPAD) consortium clinical trials was used [27,28]. This was a data-driven study that arose from a pattern found in the data. The analytical plan can be found in the S1 File.

### Study population and data sources

We collected individual participant-level data from 2 multicentre clinical trials conducted in parallel in Benin (Allada, Sékou, and Attogon), Gabon (Lambaréné and Fougamou), Kenya (Siaya), Mozambique (Manhiça and Maragra), and Tanzania (Makole and Chamwino) between 2009 and 2014 (registration numbers NCT00811421, PACTR 2010020001813440, and PACTR 2010020001429343). Both trials followed the same clinical protocols and were managed by the same team. Characteristics of study sites are described in Table 1.

**Table 1:** Characteristics of study sites.

Country	Benin	Gabon	Mozambique	Tanzania	Kenya
**Sites**	• Allada • Sékou • Attogon	• Lambaréné • Fougamou	• Manhiça • Maragra	• Makole • Chamwino	• Siaya
**Malaria transmission**	Hyperendemic	Hyperendemic	Mesoendemic	Mesoendemic	Holoendemic
**High transmission season**	April–July	October–May	September–March	June–August	May–July
**Estimated proportion of *P*. *falciparum* infections among all malaria infections in the study sites**	>90%	>90%	>90%	>90%	>90%

Adapted from González and colleagues [27,28]. Data reported in this table are estimates from 2009 prior to the clinical trials performance. The 2 clinical trials ran in parallel during the same time period.

The first study (MiPPAD 1 trial) was a randomised controlled open-label trial, performed in Benin, Gabon, Mozambique, and Tanzania, that evaluated the efficacy and safety of mefloquine (MQ) compared to sulfadoxine-pyrimethamine (SP) as intermittent preventive treatment of malaria during pregnancy (IPTp) among 4,749 HIV-uninfected pregnant women [27]. The second one (MiPPAD 2 trial) was a randomised placebo-controlled trial carried out in Mozambique, Kenya, and Tanzania, among 1,071 HIV-infected pregnant women on daily cotrimoxazole prophylaxis (CTXp) that evaluated the efficacy and safety of 3 doses of IPTp with MQ plus CTX compared to CTXp alone for prevention of malaria and opportunistic infections [28]. Trial’s sample size calculations are described in detail elsewhere [27,28]. The 2 trials ran in parallel in Mozambique and Tanzania. At enrolment, all trial participants received a long-lasting insecticide-treated net (ITN) and ferrous sulphate-folic acid supplements for prevention of anaemia in pregnancy as per national guidelines. The doses were not greater than 1.5 mg/day to avoid interference with the antifolate effect of SP and facilitate malaria parasitaemia.

The study population were pregnant women of all gravidities attending their first antenatal care visit in the current pregnancy. The inclusion criteria in the 2 trials were the same except for the HIV status of the participants: (a) ≤28 gestational weeks; (b) being residents in the study area; and (c) agreeing to give birth in one of the maternity wards of the study area. No age restrictions were applied. Women were followed monthly during pregnancy at the antenatal care clinics. At the end of pregnancy, peripheral blood and placental samples were collected from the mothers and their newborns. Malaria was assessed by passive case detection during pregnancy (thick blood smear only performed in case of malaria signs and symptoms) and actively at delivery. More information on the specific malaria diagnostic technique can be found in the original trials’ publications [27,28]. Data from the intention-to-treat populations of the 2 trials were retrieved for this analysis.

### Definitions and study outcomes

The primary study outcomes were peripheral malaria parasitaemia at delivery (measured by thick blood smear), placental infection (measured by thick blood smear and histology), and number of clinical malaria episodes during pregnancy. A clinical malaria episode was defined as the presence of asexual *Plasmodium falciparum* parasites of any density in a blood smear, plus any of the following signs and symptoms suggestive of malaria: reported history of fever in the last 24 hours, axillary temperature ≥37.5°C, pallor, arthromyalgias, headache, and history of convulsions. Secondary outcomes were peripheral PCR-confirmed *P*. *falciparum* infection at delivery, anaemia at delivery (defined by haemoglobin concentration lower than 11 g/dL), and PCR-confirmed *P*. *falciparum* placental infection. The main independent variable of the analyses was the age group, categorised into adolescents (10 to 19 years) and adults (>19 years) [29].

Additional health and demographic potential confounders were used as covariates to adjust the analyses: trial arm, gravidity, literacy (able to read and write), gestational age at recruitment (determined from fundal height measurement by bimanual palpation), mid-upper arm circumference (MUAC) (measured on left arm with MUAC tape), baseline anaemia (determined using mobile HemoCue and Hemocrontrol devices in capillary blood sample), and adherence to study treatment (IPTp) (trials’ per protocol population). Season at enrolment was also used to adjust analysis using a proxy variable where we divided the duration of recruitment into 8 periods in each study site [27,28]. These covariates were selected among the available variables collected for the clinical trials, based on the original articles reporting trial results, and an assessment of factors potentially related to the exposure (age group) and the outcomes. HIV status was used to run subgroup analysis. Gravidity was used to run supplementary subgroup analysis after removing it from the list of covariates.

### Data cleaning and analysis

Observations with missing information on age and covariates were dropped (5 cases); they were less than 0.001% of the sample. Parasitaemia at delivery, placental infection, and anaemia had missing values in 12.6%, 18.3%, and 12.5% of the participants, respectively. Overall and within each country and trial, mean age of participants remained similar after dropping missing observations. PCR infection confirmation, both for peripheral parasitaemia at delivery and placental infection, was only performed in a randomly selected subsample of participants from all countries except for Tanzania [30]. This was a complete case analysis relying on a random distribution of missing data, although participants with and without data on the primary outcomes were different regarding some covariates. More information on the differences between participants with and without data on the primary outcomes can be found in S3 File (Tables A to G).

In order to control for the effect of country and HIV status in this analysis, each country included in each trial was considered as a separate sub-study. Therefore, 4 sub-studies including HIV-uninfected women (MiPPAD Trial 1: Benin, Gabon, Mozambique (I), and Tanzania (I)) and 3 sub-studies including HIV-infected women (MiPPAD Trial 2: Kenya, Mozambique (II), and Tanzania (II)) were considered in the analyses. The 2 sub-studies carried out in Tanzania were excluded from the analysis of clinical malaria incidence and parasitaemia at delivery since malaria was not detected in any of the participants. The study performed in Tanzania among HIV-infected women was also excluded from the analysis of placental malaria infection for the same reason. Kenya was dropped from the analysis of clinical malaria since no cases among adolescents were found and no statistical adjustment could be applied to allow its inclusion. The potential bias that these exclusions may cause is discussed in last section of this article.

Prevalence was calculated using frequencies for discrete variables; means and standard deviations were used for continuous variables. Incidence outcomes were estimated as cases per person-year at risk.

Data were analysed following a 2-stage individual participant data meta-analysis (IPD-MA) approach. The availability of individual participant data allowed for adjusting the analysis by potential confounders. The first stage consisted of performing multivariable regression analyses for each outcome, per study, with age group as the principal independent variable and all the covariates as adjusting factors fixed in all models. Logistic regression models were used in the case of dichotomous outcomes, and Firth correction was applied due to low prevalence of some study outcomes [31]. Negative binomial models were chosen for the analysis of count outcomes. In the second stage, all regression model results were pooled in a standard DerSimonian–Laird random-effects model, per study outcome [32]. Subgroup analysis was carried out by HIV status of women. Heterogeneity was assessed by calculating the I^2^ statistic [33].

Supplementary sensitivity analyses were carried out for the primary outcomes with missing information (parasitaemia at delivery and placental infection) applying inverse probability weighting in order to adjust the results for potential selection bias [34]. The probability to have non-missing data on each of the outcomes was predicted though logistic regression analysis using trial arm, gravidity, literacy, gestational age at recruitment, MUAC, and baseline anaemia as regressors. Then, the inverse of this probability was used to weight the regressions of the first step of the IPD-MA. Additional sensitivity analyses were performed removing one study at a time from the meta-analyses to assess the effect of individual studies.

A supplementary subgroup analysis by gravidity (with 2 categories, primigravid and multigravida women) was performed as a robustness check for those outcomes found to be significantly associated with age group, after removing gravidity from the list of covariates. Finally, an exploratory sub-analysis to compare malaria infection between girls ≤16 years with those aged >16 to 19 years was conducted for outcomes found to be significantly associated with adolescence. It aimed at exploring whether differences between younger and older adolescents exist in the relationship with malaria in pregnancy.

The significance level was set at 0.05. All analyses were performed using the Stata statistical program version 16 (Stata Corporation, College Station, Texas, United States of America) and the command *ipdmetan* [35].

### Ethical considerations

The protocols and informed consent/assent forms of the original trials were approved by the Ethics Committee of the Hospital Clínic of Barcelona (Spain) (IP.07.31080.002), the US Centers for Disease Control and Prevention (#5609), and the national ethics review committees of the participating countries. Minors as young as 13 years were included in the clinical trials. Their inclusion followed national guidelines. In Kenya, a separate assent form was signed by minors under 16 years and the signature of the legal guardian was required. In the other countries, they were considered mature minors since they were pregnant. The trials were conducted in accordance with the Declaration of Helsinki and the Good Clinical Practice guidelines.

This study is reported as per the Strengthening the Reporting of Observational Studies in Epidemiology (STROBE) guideline (S2 File, Checklist).

## Results

### Description of the study participants

A total of 5,804 women contributed to the analyses, 1,201 (20.7%) of whom were adolescents. A detailed description of the adolescent population in terms of specific age bands by country can be found in S3 File (Table H). In the pooled sample, HIV infection was more prevalent among adults (969 of 4,603 adults, 21.1%) than among adolescents (100 of 1,201 adolescents, 8.3%). First pregnancies were more frequent among adolescent girls; 886 of 1,201 (73.8%) adolescent mothers were primigravid compared to 595 of 4,603 (12.9%) pregnant adults included in the analysis. Participants’ baseline characteristics and data origin figures by trial and age group are shown in Table 2. Unadjusted malaria in pregnancy prevalence and incidence figures for each trial can be found in Table 3.

**Table 2:** Characteristics of study participants by trial and age group.

Variables		MiPPAD 1 *N = 4*,*735*		MiPPAD 2 *N = 1*,*069*	
		≤19 years *n = 1*,*101 (23*.*3%)*	>19 years *n = 3*,*634 (76*.*7%)*	≤19 years *n = 100 (9*.*4%)*	>19 years *n = 969 (90*.*6%)*
**Demographic and baseline clinical data**					
**Age, years** ^1^		17.5 (1.4)	26.8 (5.4)	17.9 (1.0)	27.6 (5.1)
**Gravidity** ^2^	Primigravidae	832 (75.6)	541 (14.9)	54 (54.0)	54 (5.6)
**Gestational age at recruitment, weeks** ^1^		19.7 (5.2)	20.4 (5.0)	20.7 (4.7)	20.0 (5.7)
**Anaemia** ^2^	<11 g/dL Hb	701 (63.7)	2,107 (58.0)	73 (73.0)	637 (65.7)
**MUAC at baseline** ^2^	≤ 22 cm	140 (12.7)	250 (6.9)	2 (2.0)	19 (2.0)
**Literacy** ^2^	Illiterate	154 (14.0)	1,288 (35.4)	15 (15.0)	187 (19.3)
**Adherent to treatment** ^2^ ^*^	Yes	778 (70.7)	2,654 (73.0)	82 (82.0)	776 (80.1)
**Data origin**					
**HIV status** ^3^	Negative	1,101 (23.3)	3,634 (76.7)	-	-
	Positive	-	-	100 (9.4)	969 (90.6)
**Study arm** ^3^	SP	364 (23.1)	1,211 (76.9)	-	-
	MQ	737 (23.3)	2,423 (76.7)	52 (9.7)	482 (90.3)
	Placebo	-	-	48 (9.0)	487 (91.0)
**Country** ^3^	Mozambique	463 (39.2)	719 (60.8)	65 (11.6)	495 (88.4)
	Gabon	355 (30.2)	819 (69.8)	-	-
	Tanzania	162 (13.5)	1,035 (86.5)	2 (4.4)	43 (95.6)
	Benin	121 (10.2)	1,061 (89.8)	-	-
	Kenya	-	-	33 (7.1)	431 (92.9)

^1^Arithmetic mean (standard deviation).

^2^n (column percentage).

^3^n (row percentage within each trial).

*Adherent to all intermittent preventive treatment doses, per protocol trial population.

Hb, hemoglobin; MiPPAD, Malaria in Pregnancy Preventive Alternative Drugs study; MQ, mefloquine; MUAC, mid-upper arm circumference; SP, sulfadoxine-pyrimethamine.

**Table 3:** Malaria in pregnancy prevalence and incidence by trial and age group (unadjusted).

Outcomes	MiPPAD 1 *N = 4*,*735*		MiPPAD 2 *N = 1*,*069*	
	≤19 years *n = 1101*	>19 years *n = 3634*	≤19 years *n = 100*	>19 years *n = 969*
**Clinical malaria cases during pregnancy** ^1^	83/371.7 (0.2)	143/1,253.4 (0.1)	2/31.7 (0.1)	22/331.7 (0.1)
**Peripheral parasitaemia at delivery** ^2^	55/972 (5.7)	96/3,135 (3.1)	5/89 (5.6)	27/874 (3.1)
**Placental malaria** ^2^	64/915 (7.0)	127/2,932 (4.3)	6/83 (7.2)	24/815 (2.9)
**Peripheral PCR-confirmed infection at delivery** ^2^	26/216 (12.0)	97/584 (16.6)	3/55 (5.5)	24/590 (4.1)
**Placental PCR-confirmed infection** ^2^	23/204 (11.3)	87/577 (15.1)	2/52 (2.9)	26/555 (4.7)
**Anaemia at delivery** ^2^	414/969 (42.7)	1,304/3,150 (41.4)	40/88 (45.5)	337/874 (38.6)

^1^Episodes/person-year (incidence).

^2^n/N (column percentage).

The N of each outcome could be different from the total sample size of the column due to missing data.

MiPPAD, Malaria in Pregnancy Preventive Alternative Drugs study; PCR, polymerase chain reaction.

### Associations of primary outcomes with adolescence

**Fig 1:**
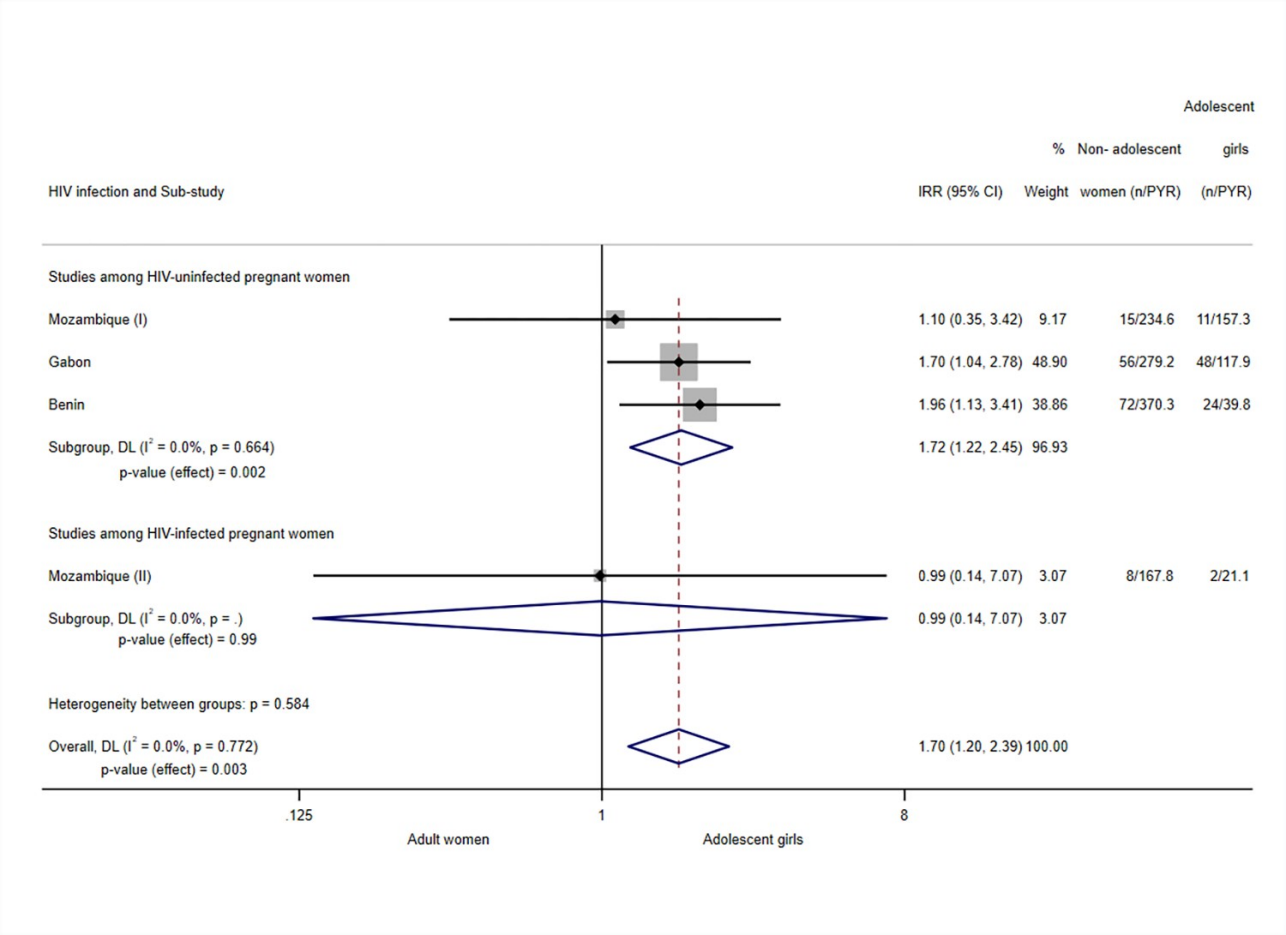
Analysis for clinical malaria episodes during pregnancy. Notes: Analyses adjusted by trial arm, gravidity, literacy, gestational age at recruitment, MUAC, anaemia at recruitment, adherence to the treatment (IPTp), and season at recruitment. Weights and between-subgroup heterogeneity test are from random-effects model. Tanzania (I), HIV-uninfected participants, and Tanzania (II), HIV-infected participants, were excluded from the analysis since no clinical malaria cases were reported in the study site. Kenya was excluded as well from the analysis because no clinical malaria episodes were reported among adolescent girls and no statistical adjustment could be applied to allow its inclusion. CI, confidence interval; DL, DerSimonian–Laird random effects model; IRR, incidence risk ratio; MUAC, mid-upper arm circumference; PYR, person-years at risk.

The incidence of clinical malaria during pregnancy was significantly higher among adolescents than among adults (incidence risk ratio (IRR) 1.70, 95% confidence interval (CI) 1.20; 2.39, *p*-value = 0.003, *N* = 4,092, 4 sub-studies) (Fig 1). There was no heterogeneity across subgroups (I^2^ = 0.0%). The analysis including only sub-studies performed among HIV-uninfected women also demonstrated a significantly higher prevalence among adolescents, with a similar magnitude (IRR 1.72, 95% CI 1.22; 2.45, *p*-value = 0.002, I^2^ = 0.0%, *N* = 3,531, 3 sub-studies), whereas the analysis among HIV-infected women did not yield statistically significant differences between adults and adolescents (IRR 0.99, 95% CI 0.14; 7.07, *p*-value = 0.99, *N* = 561, 1 sub-study).

**Fig 2:**
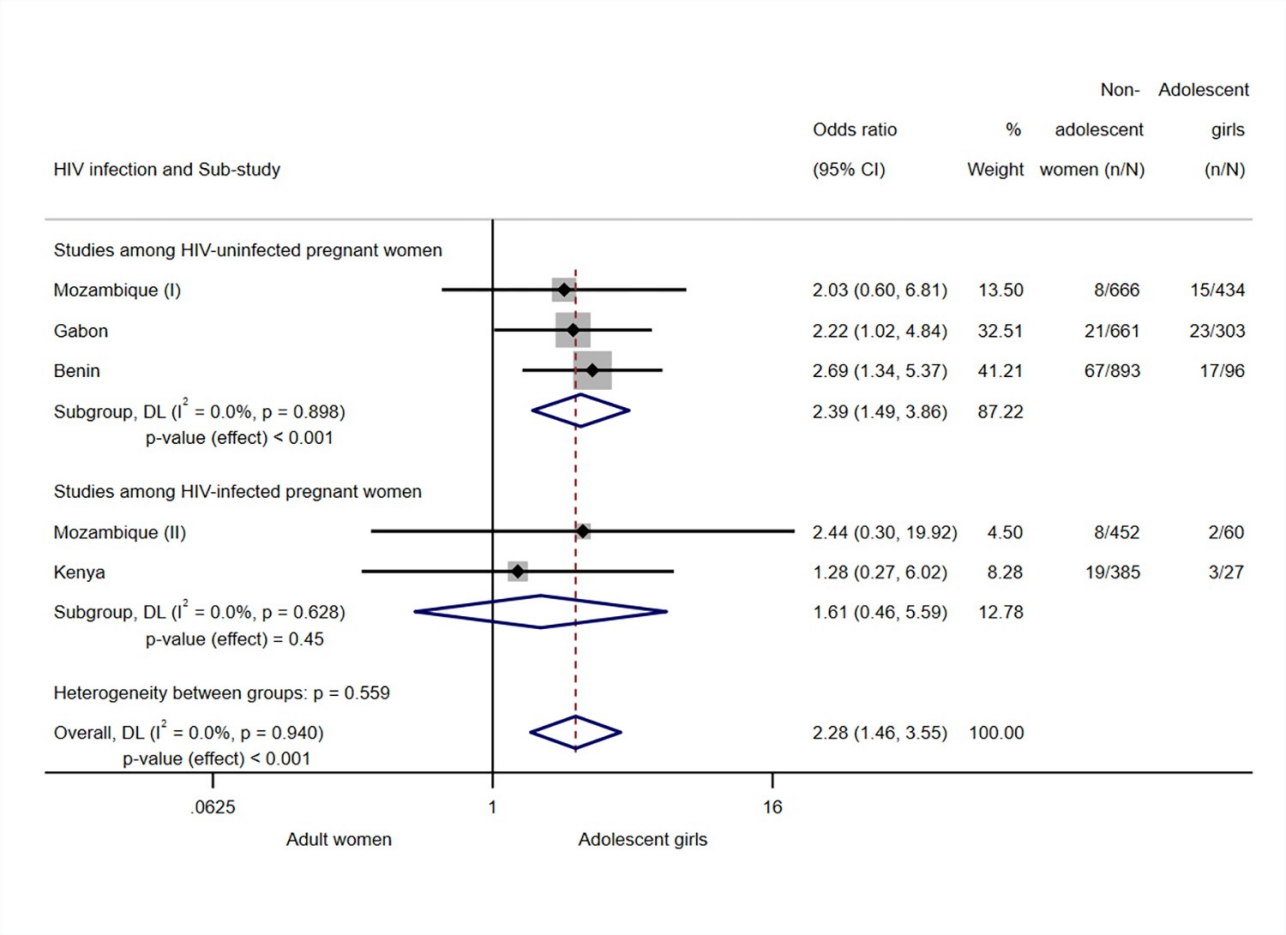
Analysis for peripheral parasitaemia at delivery. Notes: Analyses adjusted by trial arm, gravidity, literacy, gestational age at recruitment, MUAC, anaemia at recruitment, adherence to the treatment (IPTp), and season at recruitment. Weights and between-subgroup heterogeneity test are from random-effects model. Tanzania (I), HIV-uninfected participants, and Tanzania (II), HIV-infected participants, were excluded from the analysis since no cases of peripheral parasitaemia at delivery were found among study participants in the site. CI, confidence interval; DL, DerSimonian–Laird random effects model; MUAC, mid-upper arm circumference; OR, odds ratio.

The overall odds ratio (OR) for the association between age group and peripheral parasitaemia at delivery was 2.28 (95% CI 1.46; 3.55, *p*-value < 0.001, I^2^ = 0.0%, *N* = 3,977, 5 sub-studies), indicating that adolescent girls were at a greater risk for parasitaemia at delivery than adult women (Fig 2). Estimates for the subgroup analysis by HIV infection pointed to the same direction did not show heterogeneity within each group, but were only significant in the group of sub-studies among HIV-uninfected women: HIV-uninfected subgroup OR 2.39 (95% CI 1.49; 3.86, *p*-value < 0.001, I^2^ = 0.0%, *N* = 3,053, 3 sub-studies), HIV-infected subgroup OR 1.61 (95% CI 0.46; 5.59, *p*-value = 0.45, I^2^ = 0.0%, *N* = 924, 2 sub-studies).

**Fig 3:**
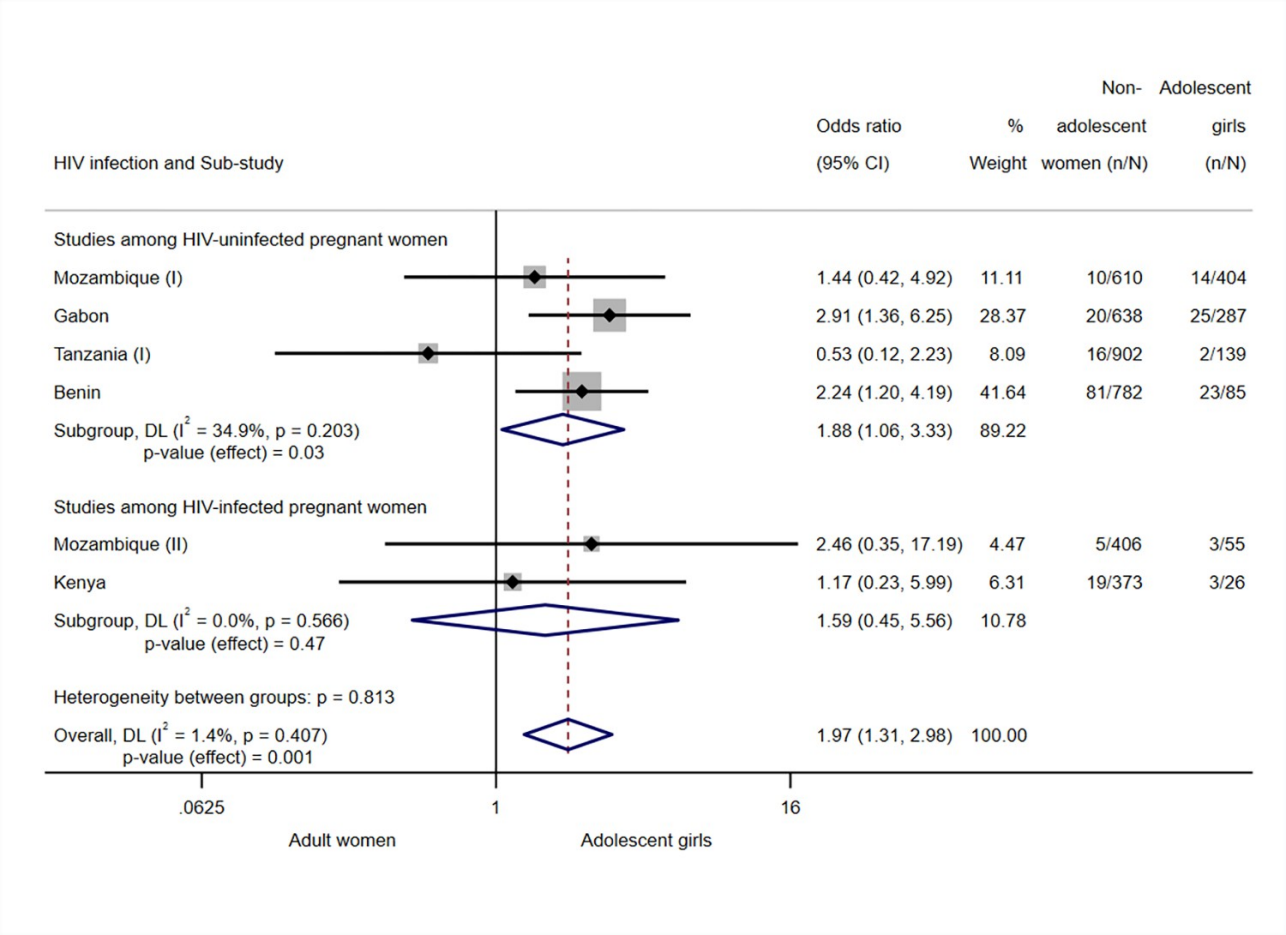
Analysis for placental malaria. Notes: Analyses adjusted by trial arm, gravidity, literacy, gestational age at recruitment, MUAC, anaemia at recruitment, adherence to the treatment (IPTp), and season at recruitment. Weights and between-subgroup heterogeneity test are from random-effects model. Tanzania (II), HIV-infected participants, was excluded from the analysis since no cases of placental infection were found among study participants in the site. CI, confidence interval; DL, DerSimonian–Laird random effects model; MUAC, mid-upper arm circumference; OR, odds ratio.

Adolescence was associated with an increased likelihood of placental infection (OR 1.97, 95% CI 1.31; 2.98, *p*-value = 0.001, I^2^ = 1.4%, *N* = 4,707, 6 sub-studies) (Fig 3). In the subgroup of studies carried out among HIV-uninfected pregnant women, the OR of the association was 1.88 (95% CI 1.06 to 3.33, *p*-value = 0.03, *N* = 3,847, 4 sub-studies), and there was moderate heterogeneity (I^2^ = 34.9%). In studies among HIV-infected women, the association observed between age and placental infection was not significant (OR 1.59, 95% CI 1.32; 5.56, *p*-value = 0.47, I^2^ = 0.0%, *N* = 860, 2 sub-studies).

### Associations of secondary outcomes with adolescence

**Fig 4:**
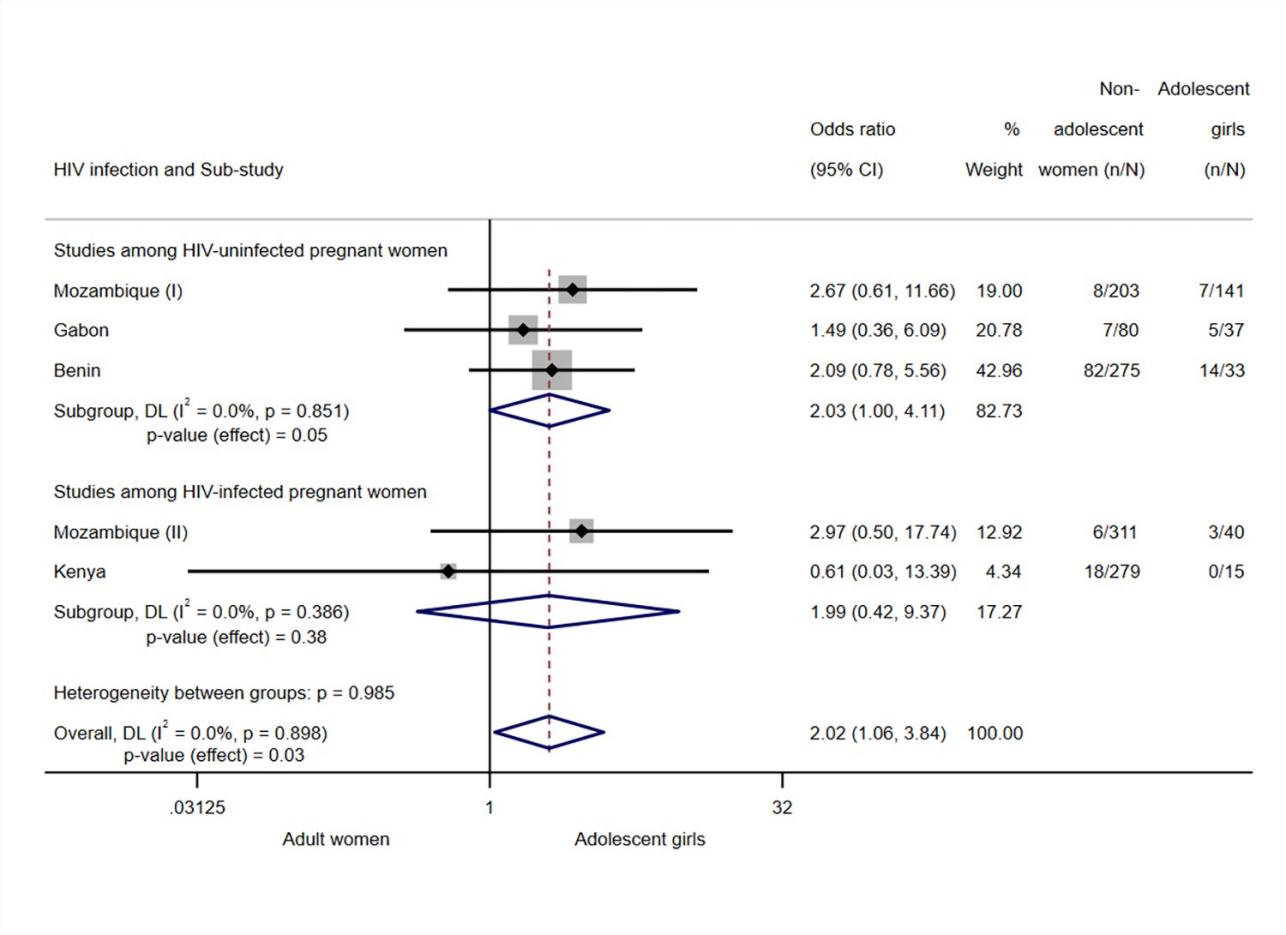
Analysis for peripheral PCR-confimed infection at delivery. Note: Analyses adjusted by trial arm, gravidity, literacy, gestational age at recruitment, MUAC, anaemia at recruitment, adherence to the treatment (IPTp), and season at recruitment. Weights and between-subgroup heterogeneity test are from random-effects model. Tanzania (I), HIV-uninfected participants, and Tanzania (II), HIV-infected participants, were excluded from the analysis since no cases of PCR-confirmed peripheral infection at delivery were found among study participants in the site. CI, confidence interval; DL, DerSimonian–Laird random effects model; MUAC, mid-upper arm circumference; OR, odds ratio.

PCR-confirmed peripheral blood malaria infection at delivery was more likely among adolescent girls than among adult women (OR 2.02, 95% CI 1.06 to 3.84, *p*-value = 0.03, I^2^ = 0.0%, *N* = 1,414, 5 sub-studies) (Fig 4).

No associations were observed between age group and prevalence of anaemia at delivery (OR 1.04, 95% CI 0.84; 1.29, *p*-value = 0.70, I^2^ = 9.8%, *N* = 5,081, 7 sub-studies) or PCR-confirmed placental infection (OR 1.45, 95% CI 0.66; 2.76, *p*-value = 0.41, I^2^ = 0.0%, *N* = 1,357, 5 sub-studies). Additional details on these analyses can be found in S3 File (Figs C and D).

### Sensitivity analysis

Regarding the sensitivity analysis where one study at a time was removed, the investigation of parasitaemia at delivery showed similar results than the primary analyses (S3 File, Table L). Conversely, the overall association between adolescence and incidence of clinical malaria during pregnancy and placental infection became nonsignificant when Benin was removed from the analysis (clinical malaria IRR 1.55, 95% CI 0.99; 2.40, *p*-value = 0.05, I^2^ = 0.0%, *N* = 2,911, 3 sub-studies; placental infection OR 1.69, 95% CI 0.91; 3.13, *p*-value = 0.10, I^2^ = 16.7%, *N* = 3,840, 5 sub-studies) (S3 File, Tables J and N). Similarly, the association between PCR-confirmed infection at delivery and adolescence became nonsignificant when Mozambique (I), Mozambique (II), or Benin were removed from the analysis (S3 File, Table P).

Applying inverse probability weighting as a sensitivity analysis for selection bias from missing data yielded similar results than primary analysis. Adolescence was found to be associated with both peripheral parasitaemia at delivery (OR 2.30, 95% CI 1.55; 3.43, *p*-value < 0.001, I^2^ = 0.0%, *N* = 3,962, 5 sub-studies) and placental malaria (OR 1.93, 95% CI 1.26; 2.95, *p*-value = 0.003, I^2^ = 18.7%, *N* = 4,442, 6 sub-studies) after applying weights. Forest plots and additional details of the sensitivity analysis can be found in S3 File (Figs A and B).

### Analyses stratified by gravidity

The results of the analyses stratified by gravidity are included in the S3 File, section 4. They showed significant associations both for primigravidae and multigravidae between adolescence and incidence of clinical malaria during pregnancy (IRR 1.74, 95% CI 1.05; 2.89, *p*-value = 0.03, I^2^ = 0.0%, *N* = 1,004, 4 sub-studies; and IRR 1.74, 95% CI 1.08; 2.80, *p*-value = 0.02, I^2^ = 0.0%, *N* = 2,593, 3 sub-studies, respectively) (Fig E in S3 File), parasitaemia at delivery (OR 2.37, 95% CI 1.16; 4.86, *p*-value = 0.02, I^2^ = 0.0%, *N* = 916, 5 sub-studies; and OR 2.01, 95% CI 1.12; 3.59, *p*-value = 0.02, I^2^ = 0.0%, *N* = 3,061, 5 sub-studies, respectively) (Fig F in S3 File) and only for multigravidae regarding placental infection (OR 2.37, 95% CI 1.35; 4.16, *p*-value = 0.003, I^2^ = 0.0% *N* = 3,498, 6 sub-studies) (Fig G in S3 File). Analyses for placental malaria among primigravidae (OR 2.74, 95% CI 0.94; 3.23, *p*-value = 0.08, I^2^ = 0.0%, *N* = 1,209, 6 sub-studies) and PCR-confirmed parasitaemia at delivery among both primigravidae and multigravidae (OR 1.85, 95% CI 0.67; 5.11, *p*-value = 0.24, I^2^ = 0.0%, *N* = 280, 5 sub-studies; OR 1.69, 95% CI 0.70; 4.08, *p*-value = 0.24, I^2^ = 0.0%, *N* = 1,134, 5 sub-studies, respectively) did not yield statistically significant associations.

### Exploratory sub-analysis among adolescents

The analyses carried out among the subsample of adolescent girls did not find significant differences between younger adolescents (≤16 years) and older adolescents (>16 years) for primary study outcomes: incidence of clinical malaria (IRR 2.02, 95% CI 0.88; 4.60, *p*-value = 0.10, I^2^ = 23.6%, *N* = 938, 3 sub-studies), parasitaemia at delivery (OR 1.33, 95% CI 0.69; 2.57, *p*-value = 0.4, I^2^ = 0.0%, *N* = 920, 5 sub-studies), and placental infection (OR 1.90, 95% CI 0.99; 3.64, *p*-value = 0.05, I^2^ = 0.0%, *N* = 996, 6 sub-studies) (Figs I and J and K in S3 File, respectively).

Detailed information on all analyses of principal and secondary outcomes, stratified analyses, sensitivity analyses, and sub-analyses, including forest plots, is provided in S3 File.

## Discussion

This study including individual participant data from over 5,800 pregnant women from 5 SSA countries found that compared to adult women, adolescents aged 13 to 19 years have increased incidence of clinical malaria during pregnancy, increased prevalence of both peripheral malaria parasitaemia at delivery, and placental infection. Associations for HIV-infected and HIV-uninfected participants were similar, but associations among HIV-infected women did not reach statistical significance. In contrast, anaemia at delivery and PCR-confirmed placental infection were not found to be more prevalent among adolescents.

The study sensitivity analyses showed that the association of adolescence with peripheral parasitaemia was not affected by the removal of single studies. Additionally, the results did not change after adjusting for selection bias using inverse probability weighting. However, the associations with incidence of clinical malaria, placental infection, and PCR-confirmed peripheral infections at delivery were no longer significant when single countries were removed from the overall analysis. This may indicate that site-specific characteristics could be important in determining the impact of age on the burden of malaria in pregnancy or just be an effect of the drop in overall sample size that would make increase the CI margins.

Since a large proportion of primigravid women in SSA are adolescents, the effect of gravidity on the association between age and malaria in pregnancy outcomes was considered. The association of adolescence with increased risk of malaria remained despite adjusting analyses by gravidity and stratifying the sample by this variable. Our results support those of a recent clinical trial performed among nulliparas that found that parasite prevalence significantly decreased with age [11].

To the best of our knowledge, this meta-analysis of individual participant data is the largest study to date investigating the association between malaria in pregnancy-related indicators among adolescent girls, compared to adult pregnant women from SSA. Moreover, we could not identify any other studies that disaggregated figures and stratified analyses by HIV infection. The comparative analysis of frequency of clinical malaria episodes between adult and adolescent pregnant women is also novel as far as we know. However, the novelty of the study examining this outcome needs to be considered in light of the fact that adolescent girls usually have asymptomatic malaria infections [24,25], and since our study may be missing asymptomatic infections during pregnancy, the comparison results are limited. Nevertheless, it is known that pregnant women report more symptoms of malaria infection than their non-pregnant peers [26], and it was unknown if those reports of malaria symptoms during pregnancy were more frequent among adolescent girls or adult women.

Our findings are in line with the studies from different SSA countries where it was observed that pregnant adolescents had increased rates of malaria parasitaemia at delivery [12–14,16]. In Gabon, a cross-sectional study conducted between 2005 and 2006 among 755 pregnant women showed that young adolescent girls (≤16 years) had significantly higher risk of malaria infection at delivery than women aged >16 years, even after performing the analyses among nulliparous women only [13]. Similarly, a study conducted between 2001 and 2002 in Tanzania among 1,684 pregnant women found that the prevalence of parasitaemia at delivery decreased with age, even when stratifying the sample by gravidity [14]. Additionally, 2 studies observed associations between adolescent pregnancies and increased placental malaria rates, although one of them did not adjust the analyses by potential confounders, and the other did not find an association after adjusting by several factors including gravidity [15,16]. It is worth noting that malaria in pregnancy prevalence figures found were much lower than those reported in some of the cited studies performed in Malawi, Cameroon, and Tanzania [12,14,16]. This difference may be due to the malaria prevention focus of the original trials and the efficacy of IPTp.

Different reasons could explain the observed associations between young age and increased risk of malaria in pregnancy. Malaria susceptibility during pregnancy is thought to be due to a combination of immunological and hormonal changes and the sequestration of infected erythrocytes in the intervillous placental space [36]. A plausible explanation for the results is the increased acquisition of malaria immunity with age following repeated exposure to different *Plasmodium* strains [37]. This could have an important impact on adolescent girls’ predisposition to become infected since they likely have less acquired immunity than adult women. Furthermore, among HIV-uninfected women, the magnitude of the association in countries with hyperendemic malaria transmission patterns, namely Benin and Gabon, was generally higher. In these countries, acquired immunity is more important than in the study area from Mozambique and this may lead to a much better tolerance to the infection among adults compared to adolescents [30]. An additional possible reason that explains differences among younger and older adolescents is the pubertal hormonal environment that could influence susceptibility to malaria [38]. A study carried out in Kenya in 1998 to 1999 showed that increased levels of the steroid dehydroepiandrosterone sulfate (DHEAS) in adolescent girls aged 12 to 18 years were associated with lower malaria parasite densities [38]. Nutritional status is a further factor that could potentially explain the observed associations. Increased levels of iron deficiency may be protective against malaria infection [39,40], and young mothers tend to present low prevalence of iron deficiency probably since they are in the post-menarcheal period and have not yet experienced iron loss due to menstruation [39].

In a previous secondary analysis of the trial among HIV-uninfected women (MiPPAD 1), very young maternal age (≤16 years) was associated with higher risk of preterm birth and low birth weight compared to older women, even after adjusting for parity [41]. This finding could be explained by the increased risk of malaria among young adolescents, but it could also be attributed to other factors affecting maternal health of adolescent girls such as biological immaturity or social and behavioural factors associated with young ages. Reduced use of ITNs has been related to young ages [42]; however, in MiPPAD trials this factor is probably not influencing the results since all women received an ITN at enrolment and no differences on net use were observed among age groups.

Regarding anaemia at delivery and contrary to our results, a previous cross-sectional study carried out in Ghana observed that adolescent girls were more likely to suffer from anaemia during pregnancy [23]. The most probable explanation for the lack of association observed is the fact that anaemia at baseline was included as a covariate in the analyses. Additionally, all study women received prophylactic ferrous sulphate at monthly antenatal clinic visits and were treated for anaemia when diagnosed, following national antenatal care guidelines [27,28]. Furthermore, anaemia has a multiplicity of contributing causes other than malaria including nutritional deficiencies, other parasitic infections, HIV, or genetic disorders [43].

Our findings also suggest that adolescent girls might experience higher risk of malaria in pregnancy regardless of their HIV status, although associations between adolescence and malaria-related outcomes for HIV-infected participants only did not reach statistical significance. To our knowledge, this is the first time this is evaluated. In this same line, our team has recently shown that the associations between age, maternal morbidity, and adverse pregnancy outcomes were not modified by HIV status among pregnant mothers from Mozambique [44]. Both the efficacy of IPTp and antiretroviral treatments for HIV-infected participants could be the reasons why we did not observe an increased malaria risk among HIV-infected individuals.

Some study limitations need to be acknowledged. First, the extrapolation of our results to other contexts should be done with caution. Our results regarding HIV-stratified analyses may not be applicable to HIV-infected populations with lower rates of treatment adherence, and the main study findings need to be interpreted in the context of antimalarials. Then, participants in clinical trials regularly attend health facilities, and consequently their health is closely monitored. Also, their background characteristics might differ from those of the general population in their countries, and their attitude towards the health system may also be different [45]. Moreover, adolescent pregnancies could carry stigma in some cultures, and it is possible that some pregnant girls do not attend antenatal care clinics. Thus, outside the clinical trial context, it is possible that adolescent girls would be at even greater risk of malaria during pregnancy compared to adult women, and in consequence, our results may be underestimating the problem. Nevertheless, the general finding of adolescents being at increased risk of malaria in pregnancy holds regardless of this limitation.

Another study limitation was the exclusion of certain countries from specific analyses. The sub-studies from Tanzania were excluded from some of the meta-analyses since they did not present cases of parasitaemia at delivery or clinical malaria episodes during pregnancy. It is worth noting that these were not true stand-alone studies, but subsamples of clinical trials. Thus, the sample size of each might have been insufficient to observe some of the outcomes by age group. This is especially applicable to HIV-infected participants, who represented only a small proportion of the total study sample. For instance, the low proportion of adolescent girls in Kenya did not allow its inclusion in the analysis of clinical malaria cases and this could have biased the results for that outcome. However, the sample size of Kenya sub-study was lower than that of other countries; therefore, its weight in the analysis would have been low. Besides, the sensitivity and stratified analyses showed consistent associations of similar magnitude and direction regardless of the studies included in the specific meta-analysis.

Then, it is necessary to acknowledge the considerable amount of missing data with regard to the study outcomes. This was mainly due lost to follow-up trial participants and an incomplete assessment of malaria outcomes among the final trial sample. Nevertheless, sensitivity analysis where inverse probability weighting was applied indicates that the associations observed still hold after accounting for potential selection bias. It is important to note that this approach relies on the correct specification of the model that includes all possible covariates associated with missingness of outcome data.

Finally, some factors and covariates known to be strongly associated with malaria risk, namely nutritional status, supplementation, and habits were not considered in this study due to the secondary nature of the data. Also, incidence of asymptomatic malaria infection during pregnancy was not analysed as a study outcome due to the study design of the original clinical trials, although it would have provided an added value to the study. Importantly, the presented findings do not allow ascertaining whether adolescent girls are at a higher risk of being infected with malaria during the course of pregnancy or if asymptomatic infections are more frequent among adolescents or adult women.

Despite these limitations, the present study includes individual participant data of more than 5,800 pregnant women from 5 different SSA countries, recruited and followed-up using the same procedures and with the same definitions of study outcomes.

Our results highlight the need for more malaria in pregnancy research focused on adolescent girls. First, malaria studies should present results by age group when possible. It is of a high priority to determine which are the mediators of the increased risk of malaria in pregnancy among adolescents and whether there are confounders that have not yet been identified. In addition, it is also fundamental to understand the health-seeking behaviour of adolescents in order to design strategies that could best target them.

From the public health perspective, the present findings indicate that there is a need to promote interventions aiming to protect adolescents from malaria. Adolescent girls should be particularly targeted in malaria control strategies including ITN distribution campaigns, even before they become pregnant. This is especially relevant because they sometimes carry asymptomatic and chronic infections before conception, and drug-based preventive strategies cannot be initiated before the second trimester of pregnancy. In addition, interventions to prevent malaria among adolescents once they become pregnant should be developed considering their specific needs.

This study is not the first calling for action to overcome the gaps in research and implementation with regard to malaria control among adolescents [46]. In 2017, the World Health Organization “Global Accelerated Action for the Health of Adolescents” programme set pregnancy care and prevention of infectious diseases among their priority areas to improve adolescents’ health [47]. However, and despite being a public health problem that has been highlighted since the early 2000s, there is still a need to take action.

In conclusion, the findings of this study support an increased likelihood of adolescent girls to experience malaria in pregnancy compared to their adult peers. Moreover, it showed that this association remains after stratifying populations by gravidity. There is a need to stress the importance of putting adolescents in the malaria research agenda and to consider them a vulnerable group to be targeted in future malaria control strategies.

## Supporting information

S1 File Analytical plan. (PDF)

S2 File STROBE checklist. (DOCX)

S3 File Additional details of the analyses. **Table A.** Characteristics of study participants with and without data on peripheral parasitaemia at delivery and placental infection in Mozambique (trial 1, HIV-uninfected participants). **Table B.** Characteristics of study participants with and without data on peripheral parasitaemia at delivery and placental infection in Mozambique (trial 2, HIV-infected participants). **Table C.** Characteristics of study participants with and without data on peripheral parasitaemia at delivery and placental infection in Gabon (trial 1, HIV-uninfected participants). **Table D.** Characteristics of study participants with and without data on peripheral parasitaemia at delivery and placental infection in Tanzania (trial 1, HIV-uninfected participants). **Table E.** Characteristics of study participants with and without data on peripheral parasitaemia at delivery and placental infection in Tanzania (trial 2, HIV-infected participants). **Table F.** Characteristics of study participants with and without data on peripheral parasitaemia at delivery and placental infection in Benin (trial 1, HIV-uninfected participants). **Table G.** Characteristics of study participants with and without data on peripheral parasitaemia at delivery and placental infection in Kenya (trial 2, HIV-infected participants). **Table H.** Number and proportion of adolescent girls by single age bands, by country. **Table I.** Number of clinical malaria cases and incidence of clinical malaria during pregnancy, by sub-study. **Table J.** Sensitivity analysis: clinical malaria incidence during pregnancy, effect of removing countries from the analysis. **Table K.** Number of participants with peripheral parasitaemia at delivery by sub-study. **Table L.** Sensitivity analysis: peripheral parasitaemia at delivery, effect of removing countries from the analysis. **Table M.** Number of participants with placental malaria by sub-study. **Table N.** Sensitivity analysis: placental malaria, effect of removing countries from the analysis. **Table O.** Number of participants with peripheral PCR-confirmed infection at delivery by sub-study. **Table P.** Sensitivity analysis: peripheral PCR-confirmed infection at delivery, effect of removing countries from the analysis. **Table Q.** Number of participants with placental PCR-confirmed malaria infection by sub-study. **Table R.** Sensitivity analysis: placental PCR-confirmed malaria infection, effect of removing countries from the analysis. **Table S.** Number of participants with anaemia at delivery by sub-study. **Table T.** Sensitivity analysis: anaemia at delivery, effect of removing countries from the analysis. **Table U.** Number of clinical malaria cases and incidence of clinical malaria during pregnancy, by sub-study and gravidity. **Table V.** Number of participants with peripheral parasitaemia at delivery by sub-study and gravidity. **Table W.** Number of participants with placental malaria by sub-study and gravidity. **Table X.** Number of participants with peripheral PCR-confirmed infection at delivery by sub-study and gravidity. **Table Y.** Number of clinical malaria cases and incidence of clinical malaria during pregnancy among adolescent girls belonging to different age groups, by sub-study. **Table Z.** Number of peripheral parasitaemia cases during pregnancy among adolescent girls belonging to different age groups, by sub-study. **Table AA.** Number of placental infection cases among adolescent girls belonging to different age groups, by sub-study. **Table AB.** Number of peripheral PCR-confirmed infections at delivery among adolescent girls belonging to different age groups, by sub-study. **Fig A.** Sensitivity analysis: peripheral parasitaemia at delivery, inverse probability weighting. **Fig B.** Sensitivity analysis: placental malaria, inverse probability weighting. **Fig C.** Analysis for placental PCR-confirmed malaria infection. **Fig D.** Analysis for anaemia at delivery. **Fig E.** Subgroup analysis by gravidity: incidence of clinical malaria during pregnancy. **Fig F.** Subgroup analysis by gravidity: peripheral parasitaemia at delivery. **Fig G.** Subgroup analysis by gravidity: placental malaria. **Fig H.** Subgroup analysis by gravidity: peripheral PCR-confirmed infection at delivery. **Fig I.** Sub-analysis among adolescent girls: incidence of clinical malaria during pregnancy. **Fig J.** Sub-analysis among adolescent girls: peripheral parasitaemia at delivery. **Fig K.** Sub-analysis among adolescent girls: placental malaria. **Fig L.** Sub-analysis among adolescent girls: peripheral PCR-confirmed infection at delivery.
(DOCX)

## Abbreviations

CI: confidence interval
CTXp: cotrimoxazole prophylaxis
DHEAS: dehydroepiandrosterone sulfate
DL: DerSimonian-Laird random-effects model
HIV: human immunodeficiency virus
IPTp: intermittent preventive treatment of malaria during pregnancy
IRR: incidence risk ratio
ITN: insecticide-treated net
IPD-MA: individual participant data meta-analysis
MiPPAD: Malaria in Pregnancy Preventive Alternative Drugs
MQ: mefloquine
MUAC: mid-upper arm circumference
OR: odds ratio
PYR: person-years at risk
SD: standard deviation
SP: sulfadoxine-pyrimethamine
SSA: sub-Saharan Africa
