# Supplementary material for: Burden of malaria in pregnancy among adolescent girls compared to adult women in 5 sub-Saharan African countries: A secondary individual participant data meta-analysis of 2 clinical trials
Source: PLoS Med. 2022 Sep 2;19(9):e1004084. doi: 10.1371/journal.pmed.1004084 (PMC9439219; doi:10.1371/journal.pmed.1004084)
Supplement: S1 File — (PDF) [file pmed.1004084.s001.pdf]

Analytical plan

Burden of malaria in pregnancy in adolescent girls from five sub-Saharan African countries

MiPPAD ancillary study

Document prepared by: Clara Pons-Duran (ISGlobal)

Revised by: Raquel González (ISGlobal), Laurence Slutsker (CDC/PATH), Valérie Briand (IRD), Michael Ramharter (BNITM), Mwaka Kakolwa (IHI).

1. Objectives

The main goal of this ancillary study is to investigate the burden of malaria in pregnancy among African adolescent girls in the context of IPTp with data from five SSA countries.

The specific objectives are:

- To determine the prevalence and incidence of malaria in pregnancy in adolescent pregnant women
- To ascertain whether adolescence was associated with malaria in pregnancy outcomes adjusting the analyses by baseline participant's characteristics

2. Methodology

Study design and data sources

This is a pooled analysis of data obtained prospectively from two clinical trials conducted in five SSA countries, between 2009 and 2014. Both trials evaluated the safety and efficacy of antimalarial drugs as IPTp. Data from the intention-to-treat cohorts will be retrieved for the analyses.

The first trial was a randomized controlled open-labelled trial, conducted in Benin, Gabon, Mozambique and Tanzania, where 4749 HIV-uninfected pregnant women were recruited to test the efficacy and safety of mefloquine (MQ) compared to SP as IPTp, and the tolerability of two dosing regimens of MQ administration in the context of use of insecticide-treated nets (ITN) (1).

The second trial was a randomised placebo-controlled trial, carried out in Mozambique, Kenya and Tanzania, where a total of 1071 HIV-infected pregnant women were recruited to evaluate the efficacy and safety of receiving three doses of IPTp with MQ, compared to placebo (2). All women received daily cotrimoxazole prophylaxis (CTXp).

The inclusion criteria of both trials were the same with the exception of the HIV status of the participants:

(a) ≤28 weeks of gestation when attending the antenatal care clinic for the first time during the current pregnancy,

(b) being residents in the study area and

(c) agreeing to give birth in one of the maternity wards of the study area.

Age was not among the inclusion or exclusion criteria, therefore women of all ages, including adolescent girls, were included in the analysis. Following WHO definition, we considered adolescents those women in the 10 to 19 years age group (3).

The selection of these two trials is purposive, and no systematic review will be performed to find additional trials. They are considered comparable in terms of inclusion criteria, study procedures, and time period of performance.

For the purpose of this pooled-analysis, each country included in each trial will be considered as a separate study; therefore, seven studies will be taken into consideration for the analyses detailed below.

Study areas

Data was collected from five SSA countries: Mozambique, Tanzania, Benin, Gabon and Kenya. Detailed information regarding the study sites can be found in the following table.

**Country**	**Benin**	**Gabon**	**Mozambique**	**Tanzania**	**Kenya**
**Sites**	• Allada<br>• Sékou<br>• Attogon	• Lambaréné<br>• Fougamou	• Manhiça<br>• Maragra	• Makole<br>• Chamwino	• Siaya
**Malaria Transmission**	Hyperendemic	Hyperendemic	Mesoendemic	Mesoendemic	Holoendemic
**High transmission season**	April-July	October-May	September-March	June-August	May-July
***P. falciparum* infection**	>90%	>90%	>90%	>90%	>90%
**# women enrolled in the trials**	1181 HIV-uninfected	1180 HIV-uninfected	1183 HIV-uninfected<br>561 HIV-infected	1200 HIV-uninfected<br>45 HIV-infected	465 HIV-infected

Adapted from González et al. 2014 I and II (1,2)

Study variables

The study outcomes considered for the analyses are malaria peripheral parasitaemia at delivery (measured by microscopic analysis of peripheral blood smear), placental infection (measured by blood smear and histology), anaemia at delivery (haemoglobin level < 11g/dL), and clinical malaria episodes during pregnancy. A clinical malaria episode is defined as the presence of asexual *Plasmodium falciparum* parasites of any density in a blood smear, plus any of the following signs and symptoms suggestive of malaria: history of fever in the last 24 hours (axillary temperature ≥37.5°C), pallor, arthromyalgias, headache and history of convulsions.

The main independent variable of the analyses will be the age group, categorized into adolescents (≤19 years), and non-adolescents (≥20 years).

The following variables of interest will be used as covariates to adjust the analyses: trial arm, compliance with IPTp, literacy, gestational age at recruitment, mid-upper arm circumference (MUAC) and baseline anaemia. HIV status and gravidity will be used to compute subgroup analyses.

Data cleaning and analysis

The databases of both trials will be merged. Observations with missing information on study outcomes, age and covariates will be dropped. It will be assessed whether women with missing information on age had different malaria outcomes than those included in the analyses, and if women with missing malaria outcomes were more likely to be adolescents.

Prevalence rates will be displayed using frequencies for discrete variables, and means and standard deviations were used for continuous variables. Incidence outcomes will be shown as cases per person-year at risk and incidence rates.

A two-stage individual patient data meta-analysis (IPD-MA) approach will be followed to analyse the pooled data. Despite using data from two clinical trials, this is an observational study, thus, standard meta-analysis techniques of intervention studies with aggregated data will not be applied. The availability of individual patient data will allow for adjusting the analysis by potential confounders (covariates mentioned above). The two-stage IPD-MA approach is commonly used when individual patient data is available but the single models are complex and the datasets large (4).

The first stage will consist on performing multivariable regression analyses for each outcome, per study, with adolescence as the principal independent variable and all the covariates as adjusting factors fixed in all models. Logistic regressions with penalized likelihood will be used in the case of dichotomous outcomes, while negative binomial models have been chosen for the analysis of count outcomes. The penalized likelihood method is used when we have complete or quasi-complete separation, or the prevalence of the outcome is low. In the context of logistic regression, this means that not all response levels are observed in each of the predictor settings.

In the second stage, all regression results will be pooled in a standard DerSimonian-Laird random-effects model, per study outcome. This widely used random-effects model accounts for between-studies heterogeneity and it is more conservative than fixed-effects models (5). Sub-group analysis will be carried out by HIV status of women. Heterogeneity will be assessed by calculating the I^2 statistic (6).

Sensitivity analyses will be performed removing one study at a time from the meta-analyses to assess the effect of individual studies. In addition, as gravidity and parity are variables usually correlated with age and they may potentially confound the analyses results, sub-group analysis by gravidity will be performed as a robustness check for those outcomes found to be significantly associated with the age group. Each study will be split into two sub-studies, separating primigravidae and multigravidae, and consequently obtaining 14 studies to be included in the meta-analysis.

The significance level is set at 0·05. All analyses will be performed using the Stata statistical program version 16 (Stata Corporation, College Station, TX, USA) (7) and the command *ipdmetan* (8).

References

1. González R, Mombo-Ngoma G, Ouédraogo S, et al. Intermittent Preventive Treatment of Malaria in Pregnancy with Mefloquine in HIV-Negative Women: A Multicentre Randomized Controlled Trial. PLoS Med 2014; 11: e1001733.
2. González R, Desai M, Macete E, et al. Intermittent preventive treatment of malaria in pregnancy with mefloquine in HIV-infected women receiving cotrimoxazole prophylaxis: a multicenter randomized placebo-controlled trial. PLoS Med 2014; 11: e1001735.
3. WHO. Adolescent health. 2020. https://www.who.int/health-topics/adolescent-health (accessed Feb 15, 2021).
4. Jackson D, Riley R, White IR. Multivariate meta-analysis: Potential and promise. Stat Med 2011; 30: 2481–98.
5. DerSimonian R, Laird N. Meta-analysis in clinical trials. Control Clin Trials 1986; 7: 177–88.
6. Higgins JPT, Thompson SG, Deeks JJ, Altman DG. Measuring inconsistency in meta-analyses. BMJ 2003; 327: 557–60.
7. StataCorp. Stata Statistical Software: Release 16. 2019.
8. Fisher D. Two-stage individual participant data meta-analysis and generalised forest plots. Stata J 2015; 15: 369–96.
