# Supplementary material for: Burden of malaria in pregnancy among adolescent girls compared to adult women in 5 sub-Saharan African countries: A secondary individual participant data meta-analysis of 2 clinical trials
Source: PLoS Med. 2022 Sep 2;19(9):e1004084. doi: 10.1371/journal.pmed.1004084 (PMC9439219; doi:10.1371/journal.pmed.1004084)
Supplement: S2 File — (DOCX) [file pmed.1004084.s002.docx]

STROBE Statement—Checklist of items that should be included in reports of ***cross-sectional studies***

|  | Item No | Recommendation | Section |
| --- | --- | --- | --- |
| **Title and abstract** | 1 | (*a*) Indicate the study’s design with a commonly used term in the title or the abstract | Title |
|  |  | (*b*) Provide in the abstract an informative and balanced summary of what was done and what was found | Abstract, authors summary |
| Introduction | | | |
| Background/rationale | 2 | Explain the scientific background and rationale for the investigation being reported | Introduction, paragraphs 1-5 |
| Objectives | 3 | State specific objectives, including any prespecified hypotheses | Introduction paragraph 6 |
| Methods | | | |
| Study design | 4 | Present key elements of study design early in the paper | Methods, Study design |
| Setting | 5 | Describe the setting, locations, and relevant dates, including periods of recruitment, exposure, follow-up, and data collection | Methods, Study population and data sources, paragraph 1 and Table 1 |
| Participants | 6 | (*a*) Give the eligibility criteria, and the sources and methods of selection of participants | Methods, Study population and data sources, paragraph 3 |
| Variables | 7 | Clearly define all outcomes, exposures, predictors, potential confounders, and effect modifiers. Give diagnostic criteria, if applicable | Methods, Definitions and study outcomes, paragraphs 1 and 2 |
| Data sources/ measurement | 8* | For each variable of interest, give sources of data and details of methods of assessment (measurement). Describe comparability of assessment methods if there is more than one group | Methods, Study population and data sources, paragraph 2  Methods, Definitions and study outcomes, paragraphs 1 and 2 |
| Bias | 9 | Describe any efforts to address potential sources of bias | Methods, Data cleaning and analysis, paragraphs 5 and 6 |
| Study size | 10 | Explain how the study size was arrived at | Methods, Study population and data sources, paragraph 2 |
| Quantitative variables | 11 | Explain how quantitative variables were handled in the analyses. If applicable, describe which groupings were chosen and why | Methods, Definitions and study outcomes, paragraphs 1 and 2  Methods, Data cleaning and analysis, paragraphs 2 and 3 |
| Statistical methods | 12 | (*a*) Describe all statistical methods, including those used to control for confounding | Methods, Data cleaning and analysis, paragraphs 3-5 |
|  |  | (*b*) Describe any methods used to examine subgroups and interactions | Methods, Data cleaning and analysis, paragraphs 3-5 |
|  |  | (*c*) Explain how missing data were addressed | Methods, Data cleaning and analysis, paragraphs 1 and 5 |
|  |  | (*d*) If applicable, describe analytical methods taking account of sampling strategy | Not applicable |
|  |  | (*e*) Describe any sensitivity analyses | Methods, Data cleaning and analysis, paragraphs 5 and 6 |
| Results | | | |
| Participants | 13* | (a) Report numbers of individuals at each stage of study—eg numbers potentially eligible, examined for eligibility, confirmed eligible, included in the study, completing follow-up, and analysed | Results, Description of study participants, paragraph 1  Original trials’ articles |
|  |  | (b) Give reasons for non-participation at each stage | Not applicable |
|  |  | (c) Consider use of a flow diagram | Not applicable |
| Descriptive data | 14* | (a) Give characteristics of study participants (eg demographic, clinical, social) and information on exposures and potential confounders | Results, Description of study participants, paragraph 1  Results, Table 2 |
|  |  | (b) Indicate number of participants with missing data for each variable of interest | Methods, Data cleaning and analysis, paragraph 1  Supplementary material S3 |
| Outcome data | 15* | Report numbers of outcome events or summary measures | Results, Table 3 |
| Main results | 16 | (*a*) Give unadjusted estimates and, if applicable, confounder-adjusted estimates and their precision (eg, 95% confidence interval). Make clear which confounders were adjusted for and why they were included | Results, Associations of primary outcome with adolescence and Associations of secondary outcomes with adolescence, Figures 1-4  Supplementary material S3 |
|  |  | (*b*) Report category boundaries when continuous variables were categorized | Not applicable |
|  |  | (*c*) If relevant, consider translating estimates of relative risk into absolute risk for a meaningful time period | Not applicable |
| Other analyses | 17 | Report other analyses done—eg analyses of subgroups and interactions, and sensitivity analyses | Results, Sensitivity analysis, Analyses stratified by gravidity and Exploratory sub-analysis among adolescents  Supplementary material S3 |
| Discussion | | | |
| Key results | 18 | Summarise key results with reference to study objectives | Discussion, paragraphs 1 and 2 |
| Limitations | 19 | Discuss limitations of the study, taking into account sources of potential bias or imprecision. Discuss both direction and magnitude of any potential bias | Discussion, paragraphs 9 to 12 |
| Interpretation | 20 | Give a cautious overall interpretation of results considering objectives, limitations, multiplicity of analyses, results from similar studies, and other relevant evidence | Discussion, paragraphs 3 to 8 |
| Generalisability | 21 | Discuss the generalisability (external validity) of the study results | Discussion, paragraphs 14 and 15 |
| Other information | | | |
| Funding | 22 | Give the source of funding and the role of the funders for the present study and, if applicable, for the original study on which the present article is based | Funding section |

*Give information separately for exposed and unexposed groups.

**Note:** An Explanation and Elaboration article discusses each checklist item and gives methodological background and published examples of transparent reporting. The STROBE checklist is best used in conjunction with this article (freely available on the Web sites of PLoS Medicine at http://www.plosmedicine.org/, Annals of Internal Medicine at http://www.annals.org/, and Epidemiology at http://www.epidem.com/). Information on the STROBE Initiative is available at www.strobe-statement.org.
