# Supplementary material for: Burden of malaria in pregnancy among adolescent girls compared to adult women in 5 sub-Saharan African countries: A secondary individual participant data meta-analysis of 2 clinical trials
Source: PLoS Med. 2022 Sep 2;19(9):e1004084. doi: 10.1371/journal.pmed.1004084 (PMC9439219; doi:10.1371/journal.pmed.1004084)
Supplement: S3 File — Table A. Characteristics of study participants with and without data on peripheral parasitaemia at delivery and placental infection in Mozambique (trial 1, HIV-uninfected participants). Table B. Characteristics of study participants with and without data on peripheral parasitaemia at delivery and placental infection in Mozambique (trial 2, HIV-infected participants). Table C. Characteristics of study participants with and without data on peripheral parasitaemia at delivery and placental infection in Gabon (trial 1, HIV-uninfected participants). Table D. Characteristics of study participants with and without data on peripheral parasitaemia at delivery and placental infection in Tanzania (trial 1, HIV-uninfected participants). Table E. Characteristics of study participants with and without data on peripheral parasitaemia at delivery and placental infection in Tanzania (trial 2, HIV-infected participants). Table F. Characteristics of study participants with and without data on peripheral parasitaemia at delivery and placental infection in Benin (trial 1, HIV-uninfected participants). Table G. Characteristics of study participants with and without data on peripheral parasitaemia at delivery and placental infection in Kenya (trial 2, HIV-infected participants). Table H. Number and proportion of adolescent girls by single age bands, by country. Table I. Number of clinical malaria cases and incidence of clinical malaria during pregnancy, by sub-study. Table J. Sensitivity analysis: clinical malaria incidence during pregnancy, effect of removing countries from the analysis. Table K. Number of participants with peripheral parasitaemia at delivery by sub-study. Table L. Sensitivity analysis: peripheral parasitaemia at delivery, effect of removing countries from the analysis. Table M. Number of participants with placental malaria by sub-study. Table N. Sensitivity analysis: placental malaria, effect of removing countries from the analysis. Table O. Number of participants with [file pmed.1004084.s003.docx]

# S3 File

## **Additional details of the analyses**

Table of contents

[1. Characteristics of study participants with and without data on peripheral parasitaemia at delivery and placental infection 2](#_Toc100993940)

[2. Age distribution of adolescent girls 5](#_Toc100993941)

[3. Details of principal analyses 6](#_Toc100993942)

[Primary outcome: Clinical malaria episodes during pregnancy 6](#_Toc90206817)

[Primary outcome: Peripheral parasitaemia at delivery 7](#_Toc90206818)

[Primary outcome: Placental malaria 9](#_Toc90206819)

[Secondary outcome: Peripheral PCR-confirmed infection at delivery 11](#_Toc90206820)

[Secondary outcome: Placental PCR-confirmed infection 12](#_Toc90206821)

[Secondary outcome: Anaemia at delivery 14](#_Toc90206822)

[4. Details of subgroup analyses by gravidity 16](#_Toc100993943)

[Primary outcome: Clinical malaria episodes during pregnancy 16](#_Toc90206824)

[Primary outcome: Peripheral parasitaemia at delivery 18](#_Toc90206825)

[Primary outcome: Placental malaria 20](#_Toc90206826)

[Secondary outcome: Peripheral PCR-confirmed infection at delivery 22](#_Toc90206827)

[5. Details of sub-analyses among adolescent girls (≤ 16 years vs >16-19 years old) 24](#_Toc100993944)

[Primary outcome: Clinical malaria episodes during pregnancy 24](#_Toc90206829)

[Primary outcome: Peripheral parasitaemia at delivery 25](#_Toc90206830)

[Primary outcome: Placental malaria 26](#_Toc90206831)

[Secondary outcome: Peripheral PCR-confirmed infection at delivery 27](#_Toc90206832)

### 1. Characteristics of study participants with and without data on peripheral parasitaemia at delivery and placental infection

Note: all participants had data on clinical malaria episodes.

Table A. Characteristics of study participants with and without data on peripheral parasitaemia at delivery and placental infection in Mozambique (trial 1, HIV-uninfected participants).

| **Variables** | | **Available data on peripheral parasitaemia AND placental infection** | **Missing data on peripheral parasitaemia OR placental infection** | **p-value** |
| --- | --- | --- | --- | --- |
|  |  | *N=1010* | *N=172* |  |
| **Age, years^1^** | | 23.1 (6.6) | 24.0 (7.1) | 0.10 |
| **Gravidity^2^** | Primigravidae | 370 (36.6) | 56 (32.6) | 0.30 |
| **Gestational age at recruitment, weeks^1^** | | 21.1 (5.0) | 20.1 (5.4) | 0.01 |
| **Anaemia^2^** | <11 g/dL Hb | 558 (55.3) | 90 (52.3) | 0.48 |
| **MUAC at baseline^2^** | <22 cm | 27 (2.7) | 8 (4.7) | 0.16 |
| **Literacy^2^** | Illiterate | 182 (18.0) | 37 (21.5) | 0.28 |
| **Adherent to treatment^2^** | | 788 (78.0) | 60 (34.88) | <0.001 |
| **Study arm^2^** | SP | 330 (32.7) | 61 (35.5) | 0.47 |
|  | MQ | 680 (67.3) | 111 (64.5) |  |

1: Arithmetic mean (standard deviation) / Mean difference independent group t-test

2: n (column percentage) / Pearson chi-squared p-value

MQ – Mefloquine; MUAC – mid-upper arm circumference; SP – sulfadoxine-pyrimethamine

Table B. Characteristics of study participants with and without data on peripheral parasitaemia at delivery and placental infection in Mozambique (trial 2, HIV-infected participants).

| **Variables** | | **Available data on peripheral parasitaemia AND placental infection** | **Missing data on peripheral parasitaemia OR placental infection** | **p-value** |
| --- | --- | --- | --- | --- |
|  |  | *N=458* | *N=102* |  |
| **Age, years^1^** | | 26.6 (5.9) | 26.7 (6.2) | 0.89 |
| **Gravidity^2^** | Primigravidae | 54 (11.8) | 11 (10.8) | 0.77 |
| **Gestational age at recruitment, weeks^1^** | | 20.5 (5.3) | 18.9 (5.8) | 0.006 |
| **Anaemia^2^** | <11 g/dL Hb | 327 (71.4) | 73 (71.6) | 0.97 |
| **MUAC at baseline^3^** | <22 cm | 9 (2.0) | 2 (2.0) | 1.00 |
| **Literacy^2^** | Illiterate | 139 (30.4) | 30 (29.4) | 0.85 |
| **Adherent to treatment^2^** | | 393 (85.8) | 43 (42.2) | <0.001 |
| **Study arm^2^** | MQ + CTXp | 227 (49.6) | 54 (52.9) | 0.54 |
|  | Placebo + CTXp | 231 (50.4) | 48 (47.1) |  |

1: Arithmetic mean (standard deviation) / Mean difference independent group t-test

2: n (column percentage) / Pearson chi-squared p-value

3: n (column percentage) / Fisher’s exact test p-value

CTXp – cotrimoxazole prophylaxis; MQ – Mefloquine; MUAC – mid-upper arm circumference

Table C. Characteristics of study participants with and without data on peripheral parasitaemia at delivery and placental infection in Gabon (trial 1, HIV-uninfected participants).

| **Variables** | | **Available data on peripheral parasitaemia AND placental infection** | **Missing data on peripheral parasitaemia OR placental infection** | **p-value** |
| --- | --- | --- | --- | --- |
|  |  | *N=915* | *N=259* |  |
| **Age, years^1^** | | 24.3 (6.9) | 25.0 (6.9) | 0.18 |
| **Gravidity^2^** | Primigravidae | 233 (25.5) | 56 (21.6) | 0.21 |
| **Gestational age at recruitment, weeks^1^** | | 18.4 (5.8) | 18.0 (6.0) | 0.31 |
| **Anaemia^2^** | <11 g/dL Hb | 628 (68.6) | 175 (67.6) | 0.75 |
| **MUAC at baseline^2^** | <22 cm | 145 (15.9) | 31 (12.0) | 0.12 |
| **Literacy^2^** | Illiterate | 156 (17.1) | 56 (21.6) | 0.09 |
| **Adherent to treatment^2^** | | 634 (69.3) | 41 (15.8) | <0.001 |
| **Study arm^2^** | SP | 312 (34.1) | 81 (31.3) | 0.40 |
|  | MQ | 603 (65.9) | 178 (68.7) |  |

1: Arithmetic mean (standard deviation) / Mean difference independent group t-test

2: n (column percentage) / Pearson chi-squared p-value

MQ – Mefloquine; MUAC – mid-upper arm circumference; SP – sulfadoxine-pyrimethamine

Table D. Characteristics of study participants with and without data on peripheral parasitaemia at delivery and placental infection in Tanzania (trial 1, HIV-uninfected participants).

| **Variables** | | **Available data on peripheral parasitaemia AND placental infection** | **Missing data on peripheral parasitaemia OR placental infection** | **p-value** |
| --- | --- | --- | --- | --- |
|  |  | *N=1036* | *N=161* |  |
| **Age, years^1^** | | 25.3 (5.5) | 24.6 (5.1) | 0.10 |
| **Gravidity^2^** | Primigravidae | 365 (35.2) | 68 (42.2) | 0.09 |
| **Gestational age at recruitment, weeks^1^** | | 20.1 (4.3) | 19.4 (4.4) | 0.05 |
| **Anaemia^2^** | <11 g/dL Hb | 493 (47.6) | 71 (44.7) | 0.50 |
| **MUAC at baseline^2^** | <22 cm | 39 (3.8) | 14 (8.7) | 0.005 |
| **Literacy^2^** | Illiterate | 85 (8.2) | 17 (10.6) | 0.32 |
| **Adherent to treatment^2^** | | 939 (90.6) | 19 (11.8) | <0.001 |
| **Study arm^2^** | SP | 343 (33.1) | 54 (33.5) | 0.91 |
|  | MQ | 693 (66.9) | 107 (66.5) |  |

1: Arithmetic mean (standard deviation) / Mean difference independent group t-test

2: n (column percentage) / Pearson chi-squared p-value

MQ – Mefloquine; MUAC – mid-upper arm circumference; SP – sulfadoxine-pyrimethamine

Table E. Characteristics of study participants with and without data on peripheral parasitaemia at delivery and placental infection in Tanzania (trial 2, HIV-infected participants).

| **Variables** | | **Available data on peripheral parasitaemia AND placental infection** | **Missing data on peripheral parasitaemia OR placental infection** | **p-value** |
| --- | --- | --- | --- | --- |
|  |  | *N=38* | *N=7* |  |
| **Age, years^1^** | | 28.4 (4.9) | 27.9 (4.2) | 0.78 |
| **Gravidity^2^** | Primigravidae | 4 (10.5) | 2 (28.6) | 0.23 |
| **Gestational age at recruitment, weeks^1^** | | 20.3 (4.2) | 23.0 (4.0) | 0.13 |
| **Anaemia^3^** | <11 g/dL Hb | 18 (47.4) | 5 (71.4) | 0.24 |
| **MUAC at baseline^2^** | <22 cm | 3 (7.9) | 0 (0) | 1.00 |
| **Literacy^2^** | Illiterate | 7 (18.4) | 2 (28.6) | 0.61 |
| **Adherent to treatment^2^** | | 34 (89.5) | 0 (0) | <0.001 |
| **Study arm^2^** | MQ + CTXp | 19 (50.0) | 3 (42.9) | 1.00 |
|  | Placebo + CTXp | 19 (50.0) | 4 (57.1) |  |

1: Arithmetic mean (standard deviation) / Mean difference independent group t-test

2: n (column percentage) / Fisher’s exact test p-value

3: n (column percentage) / Pearson chi-squared p-value

CTXp – cotrimoxazole prophylaxis; MQ – Mefloquine; MUAC – mid-upper arm circumference

Table F. Characteristics of study participants with and without data on peripheral parasitaemia at delivery and placental infection in Benin (trial 1, HIV-uninfected participants).

| **Variables** | | **Available data on peripheral parasitaemia AND placental infection** | **Missing data on peripheral parasitaemia OR placental infection** | **p-value** |
| --- | --- | --- | --- | --- |
|  |  | *N=855* | *N=327* |  |
| **Age, years^1^** | | 25.7 (5.4) | 25.8 (5.9) | 0.78 |
| **Gravidity^2^** | Primigravidae | 151 (17.7) | 74 (22.6) | 0.05 |
| **Gestational age at recruitment, weeks^1^** | | 21.9 (4.1) | 21.1 (4.7) | 0.004 |
| **Anaemia^2^** | <11 g/dL Hb | 568 (66.4) | 224 (68.5) | 0.50 |
| **MUAC at baseline^2^** | <22 cm | 87 (10.2) | 39 (11.9) | 0.38 |
| **Literacy^2^** | Illiterate | 679 (79.4) | 230 (70.3) | 0.001 |
| **Adherent to treatment^2^** | | 802 (93.8) | 149 (45.6) | <0.001 |
| **Study arm^2^** | SP | 284 (33.2) | 110 (33.6) | 0.89 |
|  | MQ | 571 (66.8) | 217 (66.4) |  |

1: Arithmetic mean (standard deviation) / Mean difference independent group t-test

2: n (column percentage) / Pearson chi-squared p-value

MQ – Mefloquine; MUAC – mid-upper arm circumference; SP – sulfadoxine-pyrimethamine

Table G. Characteristics of study participants with and without data on peripheral parasitaemia at delivery and placental infection in Kenya (trial 2, HIV-infected participants).

| **Variables** | | **Available data on peripheral parasitaemia AND placental infection** | **Missing data on peripheral parasitaemia OR placental infection** | **p-value** |
| --- | --- | --- | --- | --- |
|  |  | *N=394* | *N=70* |  |
| **Age, years^1^** | | 26.9 (5.4) | 25.5 (4.8) | 0.05 |
| **Gravidity^2^** | Primigravidae | 30 (7.6) | 7 (10.0) | 0.50 |
| **Gestational age at recruitment, weeks^1^** | | 20.0 (5.8) | 19.3 (6.2) | 0.36 |
| **Anaemia^2^** | <11 g/dL Hb | 247 (62.7) | 40 (57.1) | 0.38 |
| **MUAC at baseline^3^** | <22 cm | 5 (1.3) | 2 (2.9) | 0.29 |
| **Literacy^2^** | Illiterate | 16 (4.1) | 8 (11.4) | 0.01 |
| **Adherent to treatment^2^** | | 367 (93.2) | 21 (30.0) | <0.001 |
| **Study arm^2^** | MQ + CTXp | 195 (49.5) | 36 (51.4) | 0.77 |
|  | Placebo + CTXp | 199 (50.5) | 24 (48.6) |  |

1: Arithmetic mean (standard deviation) / Mean difference independent group t-test

2: n (column percentage) / Pearson chi-squared p-value

3: n (column percentage) / Fisher’s exact test p-value

CTXp – cotrimoxazole prophylaxis; MQ – Mefloquine; MUAC – mid-upper arm circumference

### 2. Age distribution of adolescent girls

Table H. Number and proportion of adolescent girls by single age bands, by country

| **Age (years)** | **HIV-uninfected adolescents**  (N=1101) | | | | **HIV-infected adolescents**  (N=100) | | |
| --- | --- | --- | --- | --- | --- | --- | --- |
|  | **Country** | | | | | | |
|  | **Mozambique**  n (%) | **Gabon**  n (%) | **Tanzania**  n (%) | **Benin**  n (%) | **Mozambique**  n (%) | **Tanzania**  n (%) | **Kenya**  n (%) |
| **13** | 0 (0) | 0 (0) | 0 (0) | 1 (0.8) | 0 (0) | 0 (0) | 0 (0) |
| **14** | 18 (3.9) | 11 (3.1) | 0 (0) | 0 (0) | 0 (0) | 0 (0) | 0 (0) |
| **15** | 48 (10.4) | 27 (7.6) | 1 (0.6) | 4 (3.3) | 2 (3.1) | 0 (0) | 0 (0) |
| **16** | 100 (21.6) | 51 (14.4) | 6 (3.7) | 2 (1.7) | 6 (9.2) | 0 (0) | 2 (6.1) |
| **17** | 111 (24.0) | 70 (19.7) | 19 (11.7) | 9 (7.4) | 14 (21.5) | 0 (0) | 6 (18.2) |
| **18** | 96 (10.7) | 90 (25.4) | 74 (45.7) | 54 (44.6) | 26 (40.0) | 0 (0) | 8 (24.2) |
| **19** | 90 (19.4) | 106 (29.9) | 62 (38.3) | 51 (42.2) | 17 (26.2) | 2 (100) | 17 (51.5) |
| **Total** | 463 (100) | 355 (100) | 162 (100) | 121 (100) | 65 (100) | 2 (100) | 33 (100) |

### 3. Details of principal analyses

#### Primary outcome: Clinical malaria episodes during pregnancy

- Number of included sub-studies: 4
- Total number of observations: 4092

Table I. Number of clinical malaria cases and incidence of clinical malaria during pregnancy, by sub-study

| **Sub-study** | **Overall**  n cases/person-year (incidence) | **Adolescents**  n cases/person-year (incidence) | **Adults**  n cases/person-year (incidence) |
| --- | --- | --- | --- |
| **Mozambique (I)** | 26/391.87 (0.07) | 11/157.26 (0.07) | 15/234.61 (0.06) |
| **Mozambique (II)** | 10/188.86 (0.05) | 2/21.09 (0.09) | 8/167.77 (0.05) |
| **Gabon** | 104/397.20 (0.26) | 48/117.98 (0.41) | 56/279.22 (0.20) |
| **Benin** | 96/410.14 (0.23) | 24/39.81 (0.60) | 72/370.33 (0.19) |
| **Tanzania (I) *** | 0/425.85 (0) | 0/56.63 (0) | 0/369.23 (0) |
| **Tanzania (II) *** | 0/14.85 (0) | 0/0.62 (0) | 0/14.23 (0) |
| **Kenya *** | 14/159.49 (0.09) | 0/9.94 (0) | 14/149.56 (0.09) |

** Tanzania (I), HIV-uninfected participants, and Tanzania (II), HIV-infected participants, were excluded from the analysis since no clinical malaria cases were reported in the study site. Kenya was excluded as well from the analysis because no clinical malaria episodes were reported among adolescent girls and no statistical adjustment could be applied to allow its inclusion.*

Table J. Sensitivity analysis: clinical malaria incidence during pregnancy, effect of removing countries from the analysis

| **Sub-study** | **Overall IRR (95%CI)** | **p-value** |
| --- | --- | --- |
| Without Mozambique (I) | 1.77 (1.23-2.55) | 0.002 |
| Without Mozambique (II) | 1.72 (1.22-2.45) | 0.002 |
| Without Gabon | 1.69 (1.05-2.75) | 0.03 |
| Without Benin | 1.55 (0.99-2.40) | 0.05 |

*Reference category: adult women*

*CI – confidence interval; IRR – incidence risk ratio*

#### Primary outcome: Peripheral parasitaemia at delivery

- Number of included sub-studies: 5
- Total number of observations: 3977

Table K. Number of participants with peripheral parasitaemia at delivery by sub-study

| **Sub-study** | **Overall**  n/N (%) | **Adolescents**  n/N (%) | **Adults**  n/N (%) |
| --- | --- | --- | --- |
| **Mozambique (I)** | 23/1100 (2.09) | 15/434 (3.46) | 8/666 (1.20) |
| **Mozambique (II)** | 10/512 (1.95) | 2/60 (3.33) | 8/452 (1.77) |
| **Gabon** | 44/964 (4.56) | 23/303 (7.59) | 21/661 (3.18) |
| **Benin** | 84/989 (8.49) | 17/96 (17.71) | 67/893 (7.50) |
| **Kenya** | 22/412 (5.34) | 3/27 (11.11) | 19/385 (4.94) |
| **Tanzania (I) *** | 0/1054 (0) | 0/139 (0) | 0/915 (0) |
| **Tanzania (II) *** | 0/39 (0) | 0/2 (0) | 0/37 (0) |

*** *Tanzania (I), HIV-uninfected participants, and Tanzania (II), HIV-infected participants, were excluded from the analysis since no cases of peripheral parasitaemia at delivery were found among study participants in the site.*

Table L. Sensitivity analysis: peripheral parasitaemia at delivery, effect of removing countries from the analysis

| **Sub-study** | **Overall OR (95%CI)** | **p-value** |
| --- | --- | --- |
| Without Mozambique (I) | 2.32 (1.44-3.74) | <0.001 |
| Without Mozambique (II) | 2.27 (1.44-3.58) | <0.001 |
| Without Gabon | 2.30 (1.34-3.96) | 0.003 |
| Without Benin | 2.03 (1.13-3.62) | 0.02 |
| Without Kenya | 2.40 (1.51-3.81) | <0.001 |

*Reference category: adult women*

*CI – confidence interval; OR - odds ratio*

Figure A. Sensitivity analysis: peripheral parasitaemia at delivery, inverse probability weighting


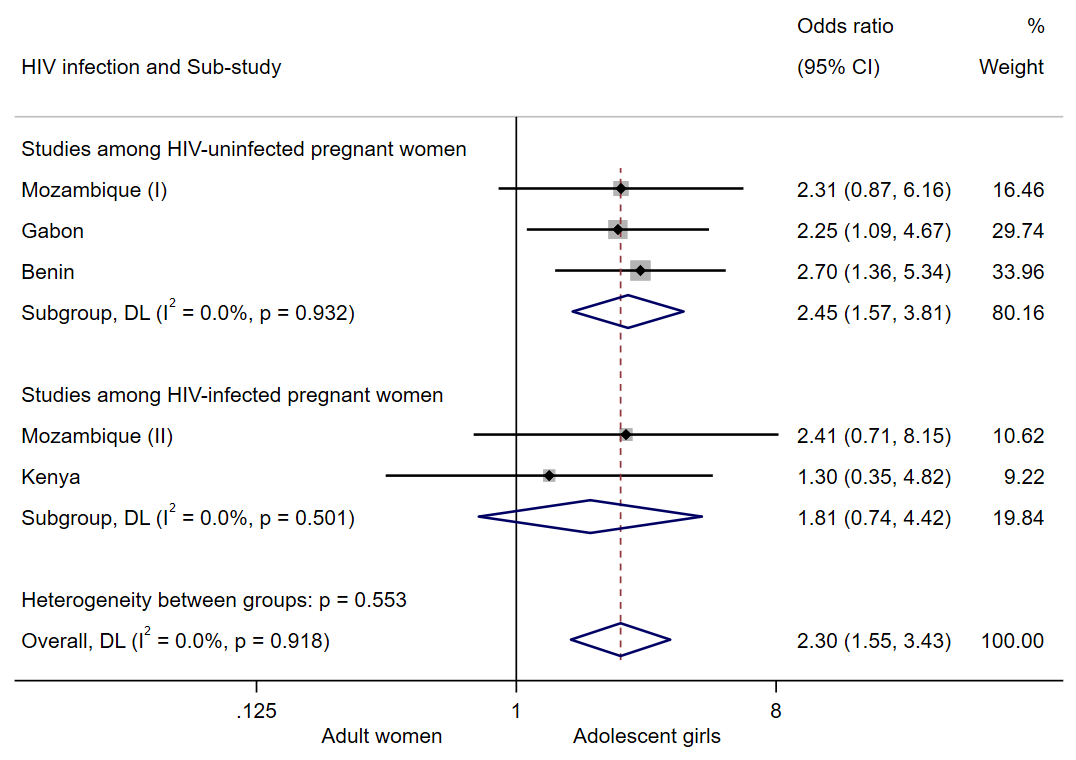


Overall p-value<0.001

HIV-uninfected participants, p-value<0.001

HIV-infected participants, p-value=0.19

*Notes: First stage of individual participant data meta-analysis based on logistic regression analysis without Firth correction. Sampling weights were applied to the regressions accounting for the probability that each participant had to be included in the analysis due to non-missing outcome data.*

*Due to data separation, 15 observations were not included in the meta-analysis, 10 from Mozambique (II) and 5 from Kenya.*

*Weights of the second stage of the meta-analysis and between-subgroup heterogeneity test are from random-effects model.*

*CI – confidence interval; DL - DerSimonian-Laird random effects model; OR – odds ratio; PCR – polymerase chain reaction*

#### Primary outcome: Placental malaria

- Number of included sub-studies: 6
- Total number of observations: 4707

Table M. Number of participants with placental malaria by sub-study

| **Sub-study** | **Overall**  n/N (%) | **Adolescents**  n/N (%) | **Adults**  n/N (%) |
| --- | --- | --- | --- |
| **Mozambique (I)** | 24/1014 (2.37) | 14/404 (3.47) | 10/610 (1.64) |
| **Mozambique (II)** | 8/461 (1.74) | 3/55 (5.45) | 5/406 (1.23) |
| **Gabon** | 45/925 (4.86) | 25/287 (8.71) | 20/638 (3.13) |
| **Tanzania (I)** | 18/1041 (1.73) | 2/139 (1.44) | 16/902 (1.77) |
| **Benin** | 104/867 (12.00) | 23/85 (27.06) | 81/782 (10.36) |
| **Kenya** | 22/399 (5.51) | 3/26 (11.54) | 19/373 (5.09) |
| **Tanzania (II) *** | 0/38 (0) | 0/2 (0) | 0/36 (0) |

** Tanzania (II), HIV-infected participants, was excluded from the analysis since no cases of placental infection were found among study participants in the site.*

Table N. Sensitivity analysis: placental malaria, effect of removing countries from the analysis

| **Sub-study** | **Overall OR (95%CI)** | **p-value** |
| --- | --- | --- |
| Without Mozambique (I) | 1.97 (1.19-3.27) | 0.009 |
| Without Mozambique (II) | 1.85 (1.13-3.04) | 0.01 |
| Without Gabon | 1.70 (1.05-2.74) | 0.03 |
| Without Tanzania (I) | 2.22 (1.45-3.39) | <0.001 |
| Without Benin | 1.69 (0.91-3.13) | 0.10 |
| Without Kenya | 1.99 (1.24-3.20) | 0.005 |

*Reference category: adult women*

*CI – confidence interval; OR – odds ratio*

Figure B. Sensitivity analysis: placental malaria, inverse probability weighting


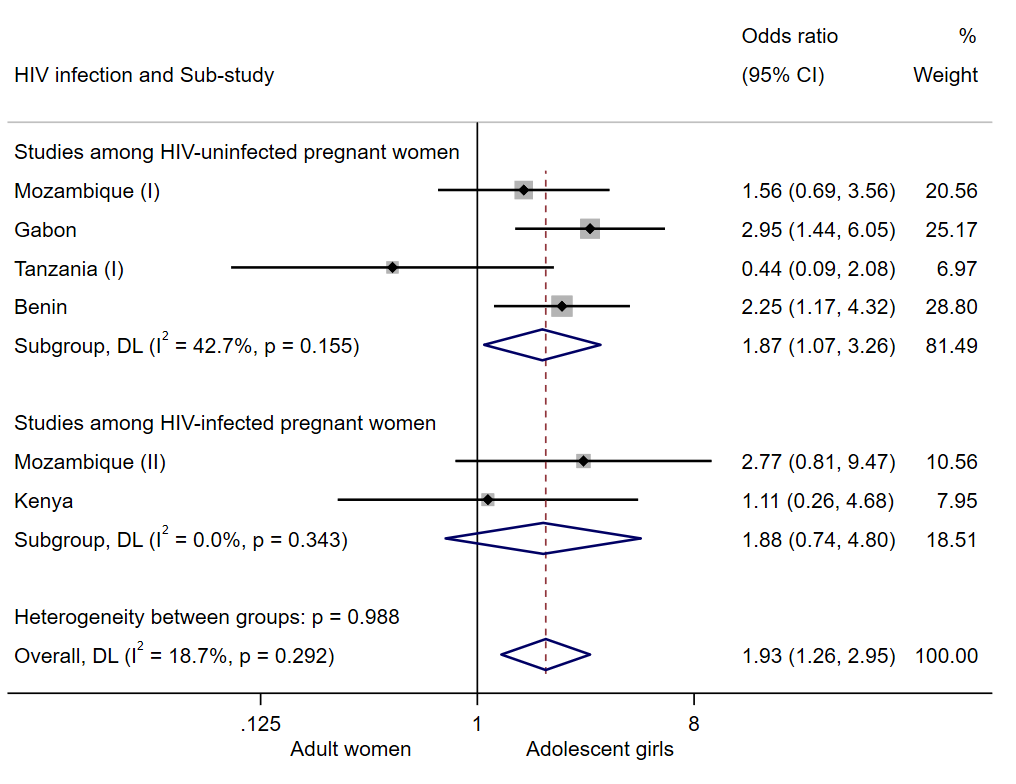


Overall p-value=0.003

HIV-uninfected participants, p-value=0.03

HIV-infected participants, p-value=0.19

*Notes: First stage of individual participant data meta-analysis based on logistic regression analysis without Firth correction. Sampling weights were applied to the regressions accounting for the probability that each participant had to be included in the analysis due to non-missing outcome data.*

*Due to data separation, 265 observations were not included in the meta-analysis, 131 from Mozambique (II), 85 from Tanzania (I) and 49 from Kenya.*

*Weights of the second stage of the meta-analysis and between-subgroup heterogeneity test are from random-effects model.*

*CI – confidence interval; DL - DerSimonian-Laird random effects model; OR – odds ratio; PCR – polymerase chain reaction*

#### Secondary outcome: Peripheral PCR-confirmed infection at delivery

- Number of included sub-studies: 5
- Total number of observations: 1414

Table O. Number of participants with peripheral PCR-confirmed infection at delivery by sub-study

| **Sub-study** | **Overall**  n/N (%) | **Adolescents**  n/N (%) | **Adults**  n/N (%) |
| --- | --- | --- | --- |
| **Mozambique (I)** | 15/344 (4.36) | 7/141 (4.96) | 8/203 (3.94) |
| **Mozambique (II)** | 9/351 (2.56) | 3/40 (7.50) | 6/311 (1.93) |
| **Gabon** | 12/117 (10.26) | 5/37 (13.52) | 7/80 (8.75) |
| **Benin** | 96/308 (31.17) | 14/33 (42.42) | 82/275 (29.82) |
| **Kenya** | 18/294 (6.12) | 0/15 (0) | 18/279 (6.45) |
| **Tanzania (I) *** | 0/31 (0) | 0/5 (0) | 0/26 (0) |
| **Tanzania (II) *** | - | - | - |

** Tanzania (I), HIV-uninfected participants, was excluded from the analysis since no cases of PCR-confirmed peripheral infection at delivery were found among study participants in the site. Tanzania (II), HIV-infected participants, was excluded since no PCR test were performed among the study participants of the site.*

*PCR – polymerase chain reaction*

Table P. Sensitivity analysis: peripheral PCR-confirmed infection at delivery, effect of removing countries from the analysis

| **Sub-study** | **Overall OR (95%CI)** | **p-value** |
| --- | --- | --- |
| Without Mozambique (I) | 1.89 (0.93-3.87) | 0.08 |
| Without Mozambique (II) | 1.91 (0.96-3.80) | 0.07 |
| Without Gabon | 2.19 (1.06-4.51) | 0.03 |
| Without Benin | 1.97 (0.84-4.62) | 0.12 |
| Without Kenya | 2.13 (1.11-4.12) | 0.02 |

*Reference category: adult women*

*CI – confidence interval; OR - odds ratio; PCR – polymerase chain reaction*

#### Secondary outcome: Placental PCR-confirmed infection

- Number of included sub-studies: 5
- Total number of observations: 1357

Table Q. Number of participants with placental PCR-confirmed malaria infection by sub-study

| **Sub-study** | **Overall**  n/N (%) | **Adolescents**  n/N (%) | **Adults**  n/N (%) |
| --- | --- | --- | --- |
| **Mozambique (I)** | 15/339 (4.42) | 7/138 (5.07) | 8/201 (3.98) |
| **Mozambique (II)** | 9/332 (2.71) | 2/37 (5.41) | 7/295 (2.37) |
| **Gabon** | 8/103 (7.77) | 5/28 (17.86) | 3/75 (4.00) |
| **Benin** | 87/308 (28.25) | 11/33 (33.33) | 76/275 (27.64) |
| **Kenya** | 19/275 (6.91) | 0/15 (0) | 19/260 (7.31) |
| **Tanzania (I)*** | 0/31 (0) | 0/5 (0) | 0/26 (0) |
| **Tanzania (II) *** | - | - | - |

** Tanzania (I), HIV-uninfected participants, was excluded from the analysis since no cases of PCR-confirmed peripheral infection at delivery were found among study participants in the site. Tanzania (II), HIV-infected participants, was excluded since no PCR test were performed among the study participants of the site.*

*PCR – polymerase chain reaction*

Figure C. Analysis for placental PCR-confirmed malaria infection


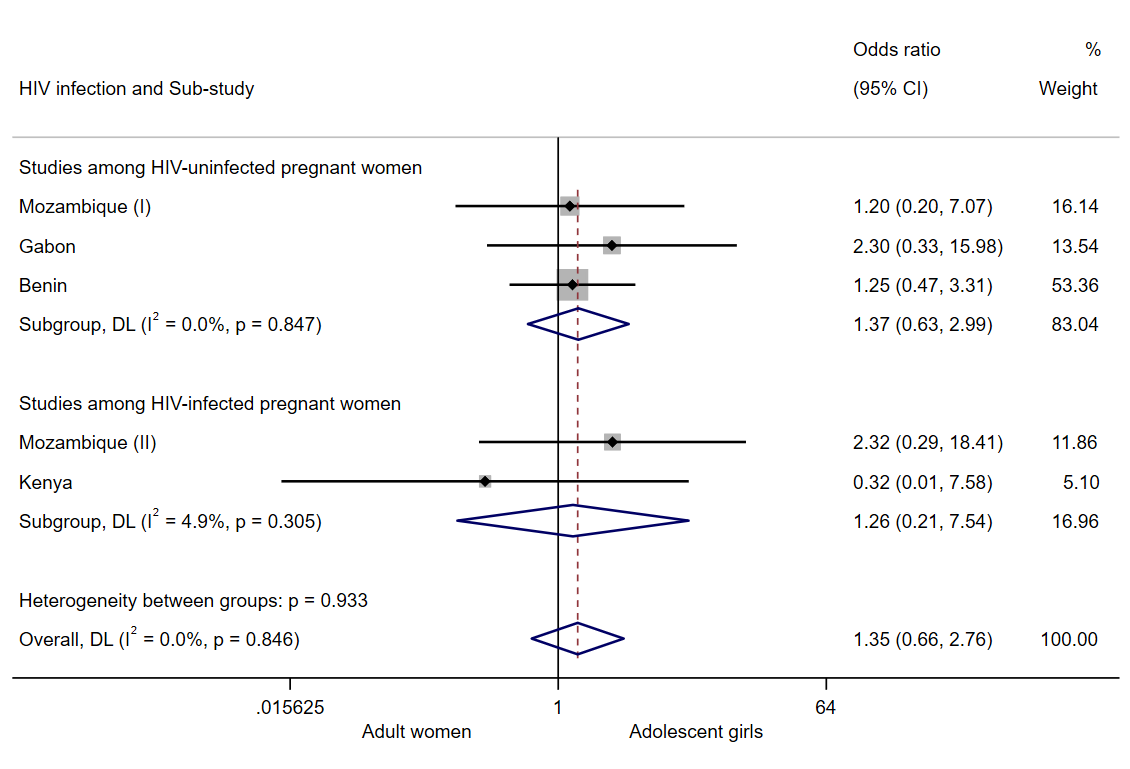


HIV-uninfected participants, p-value=0.43

HIV-infected participants, p-value=0.80

Overall, p-value=0.41

*Notes: Weights and between-subgroup heterogeneity test are from random-effects model. CI – confidence interval; DL - DerSimonian-Laird random effects model; OR – odds ratio; PCR – polymerase chain reaction*

Table R. Sensitivity analysis: placental PCR-confirmed malaria infection, effect of removing countries from the analysis

| **Study** | **Overall OR (95%CI)** | **p-value** |
| --- | --- | --- |
| Without Mozambique (I) | 1.38 (0.66-3.02) | 0.41 |
| Without Mozambique (II) | 1.26 (0.59-2.69) | 0.55 |
| Without Gabon | 1.24 (0.58-2.68) | 0.58 |
| Without Benin | 1.48 (0.52-4.22) | 0.46 |
| Without Kenya | 1.35 (0.67-2.72) | 0.40 |

*Reference category: adult women*

*CI – confidence interval; OR - odds ratio; PCR – polymerase chain reaction*

#### Secondary outcome: Anaemia at delivery

- Number of included sub-studies: 7
- Total number of observations: 5081

Table S. Number of participants with anaemia at delivery by sub-study

| **Study** | **Overall**  n/N (%) | **Adolescents**  n/N (%) | **Adults**  n/N (%) |
| --- | --- | --- | --- |
| **Mozambique (I)** | 446/1098 (40.62) | 169/434 (39.94) | 277/664 (41.72) |
| **Mozambique (II)** | 252/510 (49.41) | 32/59 (54.24) | 220/451 (48.78) |
| **Gabon** | 485/967 (50.16) | 158/300 (52.67) | 327/667 (49.03) |
| **Tanzania (I)** | 394/1057 (37.28) | 47/139 (33.81) | 347/918 (37.80) |
| **Tanzania (II)** | 19/39 (48.72) | 0/2 (0) | 19/37 (51.35) |
| **Benin** | 393/997 (39.42) | 40/96 (41.67) | 353/901 (39.18) |
| **Kenya** | 106/413 (25.67) | 8/27 (29.63) | 98/386 (25.39) |

Figure D. Analysis for anaemia at delivery


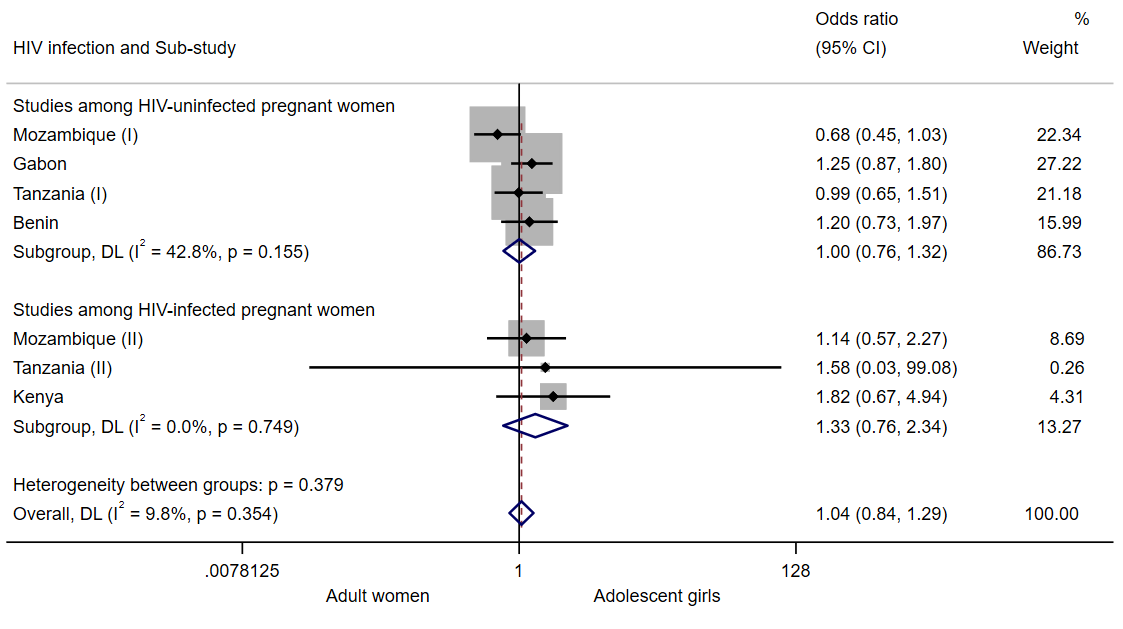


HIV-uninfected participants, p-value=0.99

HIV-infected participants, p-value=0.32

Overall, p-value=0.70

*Notes: Weights and between-subgroup heterogeneity test are from random-effects model. CI – confidence interval; DL - DerSimonian-Laird random effects model; OR – odds ratio*

Table T. Sensitivity analysis: anaemia at delivery, effect of removing countries from the analysis

| **Sub-study** | **Overall OR (95%CI)** | **p-value** |
| --- | --- | --- |
| Without Mozambique (I) | 1.00 (0.83-1.21) | 0.97 |
| Without Mozambique (II) | 0.90 (0.77-1.06) | 0.20 |
| Without Gabon | 0.85 (0.70-1.02) | 0.08 |
| Without Tanzania (I) | 0.93 (0.79-1.11) | 0.43 |
| Without Tanzania (II) | 0.92 (0.78-1.07) | 0.26 |
| Without Benin | 0.91 (0.77-1.07) | 0.26 |
| Without Kenya | 0.91 (0.78-1.07) | 0.24 |

*Reference category: adult women*

*CI – confidence interval; OR - odds ratio*

### 4. Details of subgroup analyses by gravidity

Only performed for those outcomes where significant differences were found between adolescent and adult pregnant women in the principal analysis.

#### Primary outcome: Clinical malaria episodes during pregnancy

- Number of included sub-studies: 7 (4 including primigravidae, 3 including multigravidae)
- Total number of observations: 3597

Table U. Number of clinical malaria cases and incidence of clinical malaria during pregnancy, by sub-study and gravidity

| **Sub-study** | **Primigravidae**  n cases/person-year (incidence) | | | **Multigravidae**  n cases/person-year (incidence) | | |
| --- | --- | --- | --- | --- | --- | --- |
|  | **Overall** | **Adolescents** | **Adults** | **Overall** | **Adolescents** | **Adults** |
| **Mozambique (I)** | 10/145.14 (0.07) | 9/126.69 (0.07) | 1/18.45 (0.05) | 16/246.73 (0.06) | 2/30.57 (0.07) | 14/216.16 (0.06) |
| **Gabon** | 39/98.22 (0.40) | 32/77.10 (0.42) | 7/21.12 (0.33) | 65/298.98 (0.22) | 16/40.88 (0.39) | 49/258.09 (0.19) |
| **Benin** | 37/75.41 (0.49) | 20/28.52 (0.70) | 17/46.89 (0.36) | 59/334.73 (0.18) | 4/11.29 (0.35) | 55/323.44 (0.17) |
| **Mozambique (II)*** | 3/22.12 (0.14) | 2/11.98 (0.17) | 1/10.14 (0.10) | 7/166.74 (0.04) | 0/9.11 (0) | 7/157.63 (0.04) |
| **Tanzania (I) *** | 0/151.05 (0) | 0/48.32 (0) | 0/102.72 (0) | 0/274.81 (0) | 0/8.30 (0) | 0/266.50 (0) |
| **Tanzania (II) *** | 0/1.63 (0) | - | 0/1.63 (0) | 0/13.22 (0) | 0/0.62 (0) | 0/12.59 (0) |
| **Kenya *** | 1/11.97 (0.08) | 0/5.09 (0) | 1/6.88 (0.15) | 13/147.52 (0.09) | 0/4.85 (0) | 13/142.68 (0.09) |

** Tanzania (I), HIV-uninfected participants, and Tanzania (II), HIV-infected participants, were excluded from the analysis since no clinical malaria cases were reported in the study site. Kenya and multigravidae from Mozambique (II) were excluded as well from the analysis because no clinical malaria episodes were reported among adolescent girls and no statistical adjustment could be applied to allow their inclusion.*

Figure E. Subgroup analysis by gravidity: incidence of clinical malaria during pregnancy


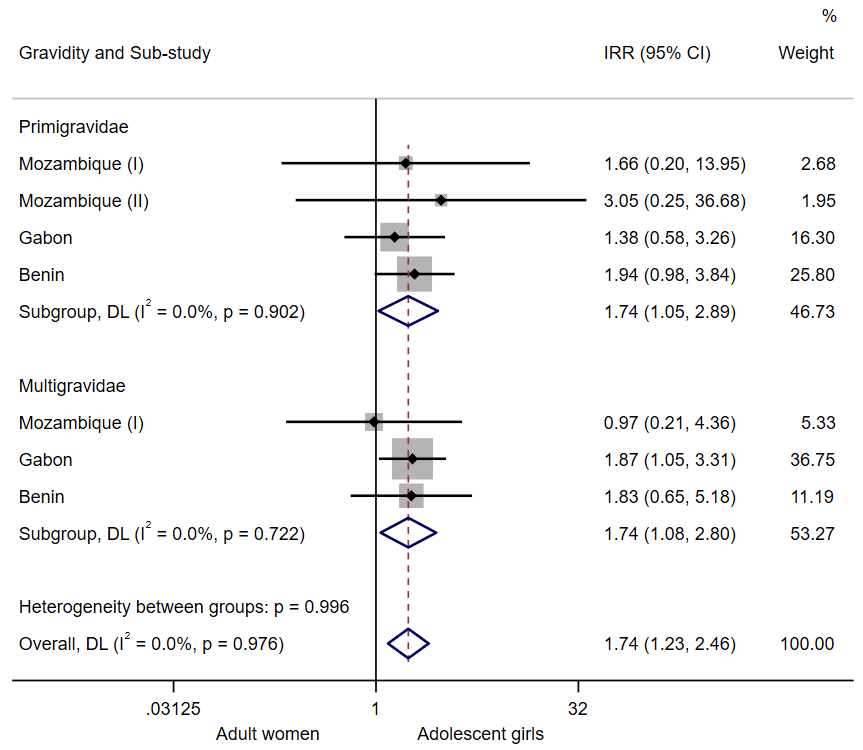


Primigravidae: p-value=0.03

Multigravidae: p-value=0.02

Overall: p-value=0.002

*Notes: Weights and between-subgroup heterogeneity test are from random-effects model. CI – confidence interval; DL - DerSimonian-Laird random effects model; IRR – incidence risk ratio*

#### Primary outcome: Peripheral parasitaemia at delivery

- Number of included sub-studies: 10 (5 including primigravidae, 5 including multigravidae)
- Total number of observations: 3977

Table V. Number of participants with peripheral parasitaemia at delivery by sub-study and gravidity

| **Sub-study** | **Primigravidae**  n/N (%) | | | **Multigravidae**  n/N (%) | | |
| --- | --- | --- | --- | --- | --- | --- |
|  | **Overall** | **Adolescents** | **Adults** | **Overall** | **Adolescents** | **Adults** |
| **Mozambique (I)** | 13/395 (3.29) | 13/348 (3.74) | 0/47 (0) | 10/705 (1.42) | 2/86 (2.33) | 8/619 (1.29) |
| **Mozambique (II)** | 2/60 (3.33) | 2/34 (5.88) | 0/26 (0) | 8/452 (1.77) | 0/26 (0) | 8/426 (1.88) |
| **Gabon** | 18/245 (7.35) | 17/200 (8.50) | 1/44 (2.27) | 26/719 (3.62) | 6/103 (5.83) | 20/616 (3.25) |
| **Benin** | 20/184 (10.87) | 12/67 (17.91) | 8/117 (6.84) | 64/805 (7.95) | 5/29 (17.24) | 59/776 (7.60) |
| **Kenya** | 5/32 (15.63) | 3/14 (21.43) | 2/18 (11.11) | 17/380 (4.47) | 0/13 (0) | 17/367 (4.63) |
| **Tanzania (I) *** | 0/369 (0) | 0/122 (0) | 0/247 (0) | 0/685 (0) | 0/17 (0) | 0/668 (0) |
| **Tanzania (II) *** | 0/5 (0) | 0/0 (0) | 0/5 (0) | 0/34 (0) | 0/2 (0) | 0/32 (0) |

** Tanzania (I), HIV-uninfected participants, and Tanzania (II), HIV-infected participants, were excluded from the analysis since no cases of peripheral parasitaemia at delivery were found among study participants in the site.*

Figure F. Subgroup analysis by gravidity: peripheral parasitaemia at delivery


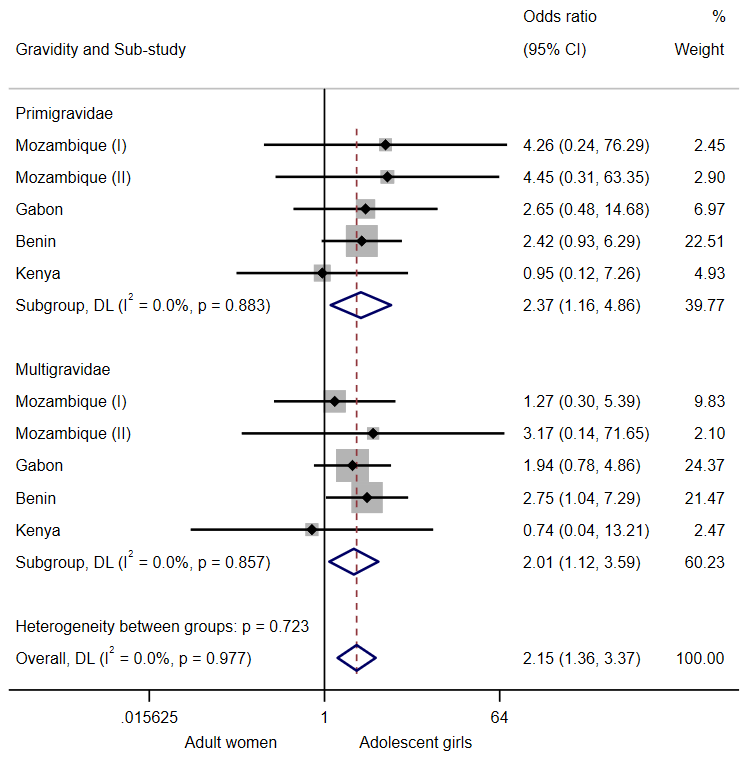


Primigravidae: p-value=0.02

Multigravidae: p-value=0.02

Overall: p-value<0.001

*Notes: Weights and between-subgroup heterogeneity test are from random-effects model. CI – confidence interval; DL – DerSimonian-Laird random effects model; OR – odds ratio*

#### Primary outcome: Placental malaria

- Number of included sub-studies: 12 (6 including primigravidae, 6 including multigravidae)
- Total number of observations: 4707

Table W. Number of participants with placental malaria by sub-study and gravidity

| **Sub-study** | **Primigravidae**  n/N (%) | | | **Multigravidae**  n/N (%) | | |
| --- | --- | --- | --- | --- | --- | --- |
|  | **Overall** | **Adolescents** | **Adults** | **Overall** | **Adolescents** | **Adults** |
| **Mozambique (I)** | 13/371 (3.50) | 13/326 (3.99) | 0/45 (0) | 11/643 (1.71) | 1/78 (1.28) | 10/565 (1.77) |
| **Mozambique (II)** | 3/54 (5.56) | 3/31 (9.68) | 0/23 (0) | 5/407 (1.23) | 0/24 (0) | 5/383 (1.31) |
| **Gabon** | 18/234 (7.69) | 17/189 (8.99) | 1/45 (2.22) | 27/691 (3.91) | 8/98 (8.16) | 19/593 (3.20) |
| **Tanzania (I)** | 9/366 (2.46) | 2/122 (1.64) | 7/244 (2.87) | 9/675 (1.33) | 0/17 (0) | 9/658 (1.37) |
| **Benin** | 32/153 (20.92) | 17/59 (28.81) | 15/94 (15.96) | 72/714 (10.08) | 6/26 (23.08) | 66/688 (9.59) |
| **Kenya** | 5/31 (16.13) | 3/14 (21.43) | 2/17 (11.76) | 17/368 (4.62) | 0/12 (0) | 17/356 (4.78) |
| **Tanzania (II) *** | 0/4 (0) | - | 0/4 (0) | 0/34 (0) | 0/2 (0) | 0/32 (0) |

** Tanzania (II), HIV-infected participants, was excluded from the analysis since no cases of placental infection were found among study participants in the site.*

Figure G. Subgroup analysis by gravidity: placental malaria


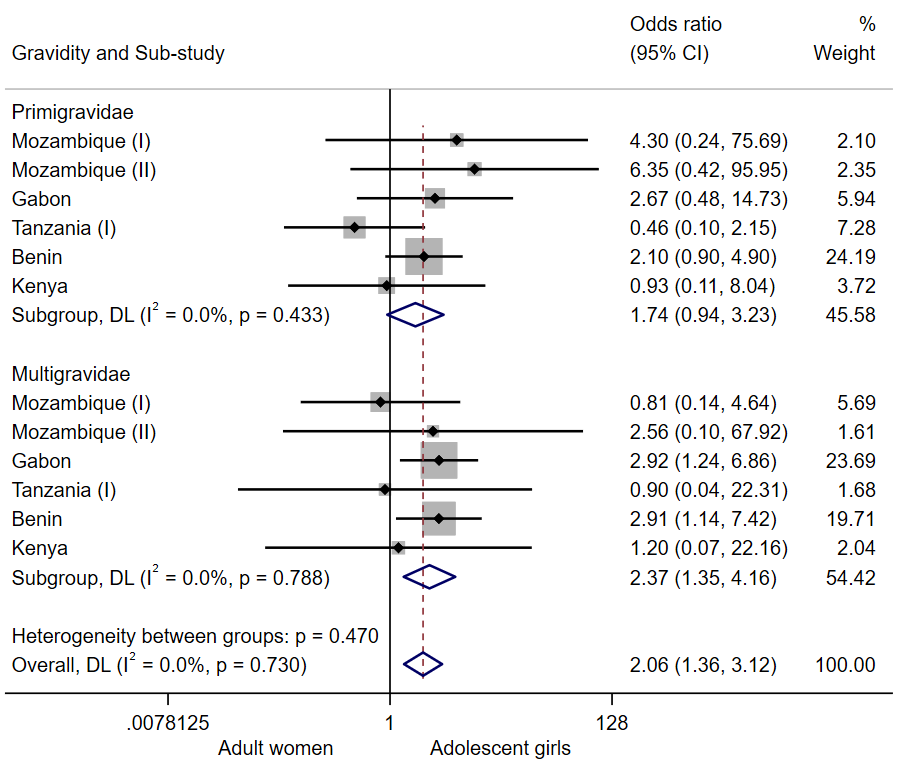


Primigravidae: p-value=0.08

Multigravidae: p-value=0.003

Overall: p-value<0.001

*Notes: Weights and between-subgroup heterogeneity test are from random-effects model. CI – confidence interval; DL – DerSimonian-Laird random effects model; OR – odds ratio*

#### Secondary outcome: Peripheral PCR-confirmed infection at delivery

- Number of included sub-studies: 10 (5 including primigravidae, 5 including multigravidae)
- Total number of observations: 1414

Table X. Number of participants with peripheral PCR-confirmed infection at delivery by sub-study and gravidity

| **Sub-study** | **Primigravidae**  n/N (%) | | | **Multigravidae**  n/N (%) | | |
| --- | --- | --- | --- | --- | --- | --- |
|  | **Overall** | **Adolescents** | **Adults** | **Overall** | **Adolescents** | **Adults** |
| **Mozambique (I)** | 5/131 (3.82) | 5/117 (4.27) | 0/14 (0) | 10/213 (4.60) | 2/24 (8.33) | 8/189 (4.23) |
| **Mozambique (II)** | 3/39 (7.69) | 3/19 (15.79) | 0/20 (0) | 6/312 (1.92) | 0/21 (0) | 6/291 (2.06) |
| **Gabon** | 5/34 (14.71) | 4/25 (16.00) | 1/9 (11.11) | 7/83 (8.43) | 1/12 (8.33) | 6/71 (8.45) |
| **Benin** | 16/59 (27.12) | 10/26 (38.46) | 6/33 (18.18) | 80/249 (32.13) | 4/7 (57.14) | 76/242 (31.40) |
| **Kenya** | 1/17 (5.88) | 0/5 (0) | 1/12 (8.33) | 17/277 (6.14) | 0/10 (0) | 17/267 (6.37) |
| **Tanzania (I) *** | 0/13 (0) | 0/3 (0) | 0/10 (0) | 0/18 (0) | 0/2 (0) | 0/16 (0) |
| **Tanzania (II) *** | - | - | - | - | - | - |

** Tanzania (I), HIV-uninfected participants, was excluded from the analysis since no cases of PCR-confirmed peripheral infection at delivery were found among study participants in the site. Tanzania (II), HIV-infected participants, was excluded since no PCR test were performed among the study participants of the site..*

*PCR – polymerase chain reaction*

Figure H. Subgroup analysis by gravidity: peripheral PCR-confirmed infection at delivery


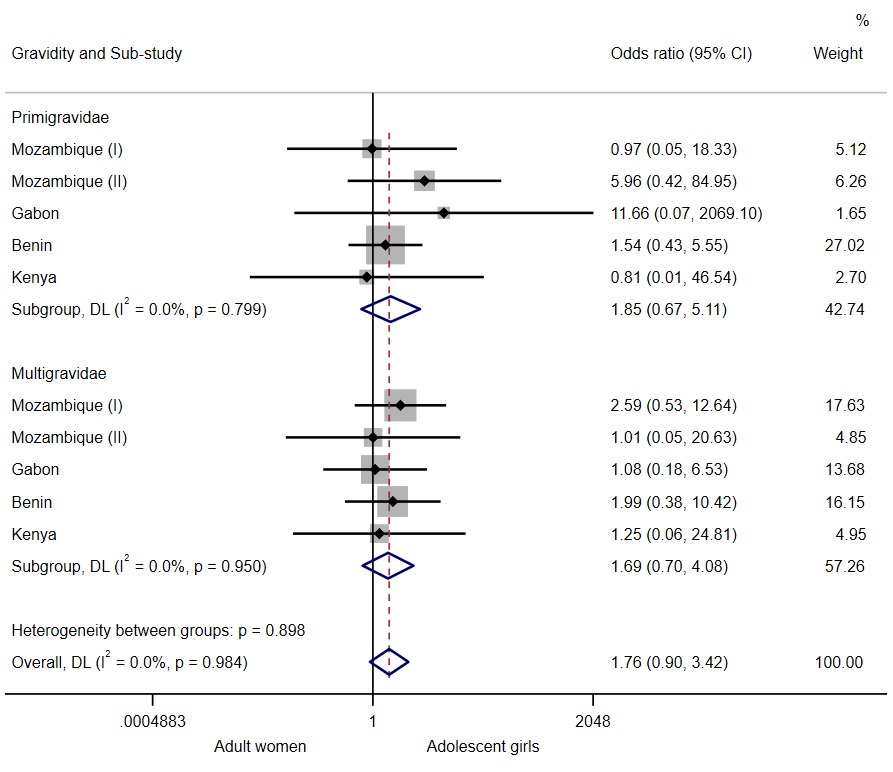


Primigravidae: p-value=0.24

Multigravidae: p-value=0.24

Overall: p-value=0.10

*Notes: Weights and between-subgroup heterogeneity test are from random-effects model. CI – confidence interval; DL – DerSimonian-Laird random effects model; OR – odds ratio; PCR – polymerase chain reaction*

### 5. Details of sub-analyses among adolescent girls (≤ 16 years vs >16-19 years old)

Only performed for those outcomes where significant differences were found between adolescent and adult pregnant women in the principal analysis.

#### Primary outcome: Clinical malaria episodes during pregnancy

- Number of included sub-studies: 3
- Total number of observations: 938

Table Y. Number of clinical malaria cases and incidence of clinical malaria during pregnancy among adolescent girls belonging to different age groups, by sub-study

| **Sub-study** | **Overall**  n cases/person-year (incidence) | **≤16 years**  n cases/person-year (incidence) | **>16-19 years**  n cases/person-year (incidence) |
| --- | --- | --- | --- |
| **Gabon** | 48/117.98 (0.41) | 17/29.41 (0.58) | 31/88.57 (0.35) |
| **Benin** | 24/39.81 (0.60) | 1/2.31 (0.43) | 23/37.50 (0.61) |
| **Mozambique (I)** | 11/157.26 (0.07) | 7/55.35 (0.13) | 4/101.91 (0.04) |
| **Mozambique (II)*** | 2/21.09 (0.09) | 0/2.75 (0) | 2/18.34 (0.11) |
| **Tanzania (I) *** | 0/56.63 (0) | 0/2.52 (0) | 0/54.10 (0) |
| **Tanzania (II) *** | 0/0.62 (0) | - | 0/0.62 (0) |
| **Kenya *** | 0/9.94 (0) | 0/0.59 (0) | 0/9.34 (0) |

** Tanzania (I), HIV-uninfected participants, and Tanzania (II), HIV-infected participants, were excluded from the analysis since no clinical malaria cases were reported in the study site. Kenya was excluded because no clinical malaria episodes were reported among adolescent girls. Mozambique (II), HIV-infected participants, was excluded because no cases of clinical malaria were reported among young adolescents ≤16 years. No statistical adjustment could be applied to allow the inclusion of Kenya and Mozambique (II).*

Figure I. Sub-analysis among adolescent girls: incidence of clinical malaria during pregnancy

*
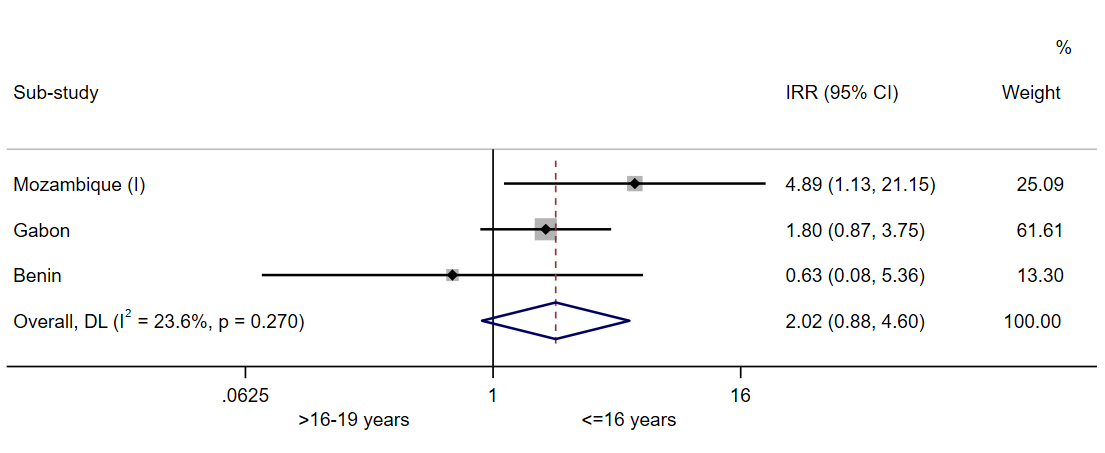
*

p-value=0.10

*Note: Weights and between-subgroup heterogeneity test are from random-effects model. CI – confidence interval; DL – DerSimonian-Laird random effects model; IRR – incidence risk ratio*

#### Primary outcome: Peripheral parasitaemia at delivery

- Number of included sub-studies: 5
- Total number of observations: 920

Table Z. Number of peripheral parasitaemia cases during pregnancy among adolescent girls belonging to different age groups, by sub-study

| **Sub-study** | **Overall**  n/N (%) | **≤16 years**  n/N (%) | **>16-19 years**  n/N (%) |
| --- | --- | --- | --- |
| **Mozambique (I)** | 15/434 (3.46) | 8/153 (5.23) | 7/281 (2.49) |
| **Mozambique (II)** | 2/60 (3.33) | 0/8 (0) | 2/52 (3.85) |
| **Gabon** | 23/303 (7.59) | 8/82 (9.76) | 15/221 (6.79) |
| **Benin** | 17/96 (17.71) | 1/6 (16.67) | 16/90 (17.78) |
| **Kenya** | 3/27 (11.11) | 0/2 (0) | 3/25 (12.00) |
| **Tanzania (I) *** | 0/139 (0) | 0/7 (0) | 0/132 (0) |
| **Tanzania (II) *** | 0/2 (0) | - | 0/2 (0) |

** Tanzania (I), HIV-uninfected participants, and Tanzania (II), HIV-infected participants, were excluded from the analysis since no cases of peripheral parasitaemia at delivery were found among study participants in the site.*

Figure J. Sub-analysis among adolescent girls: peripheral parasitaemia at delivery

*
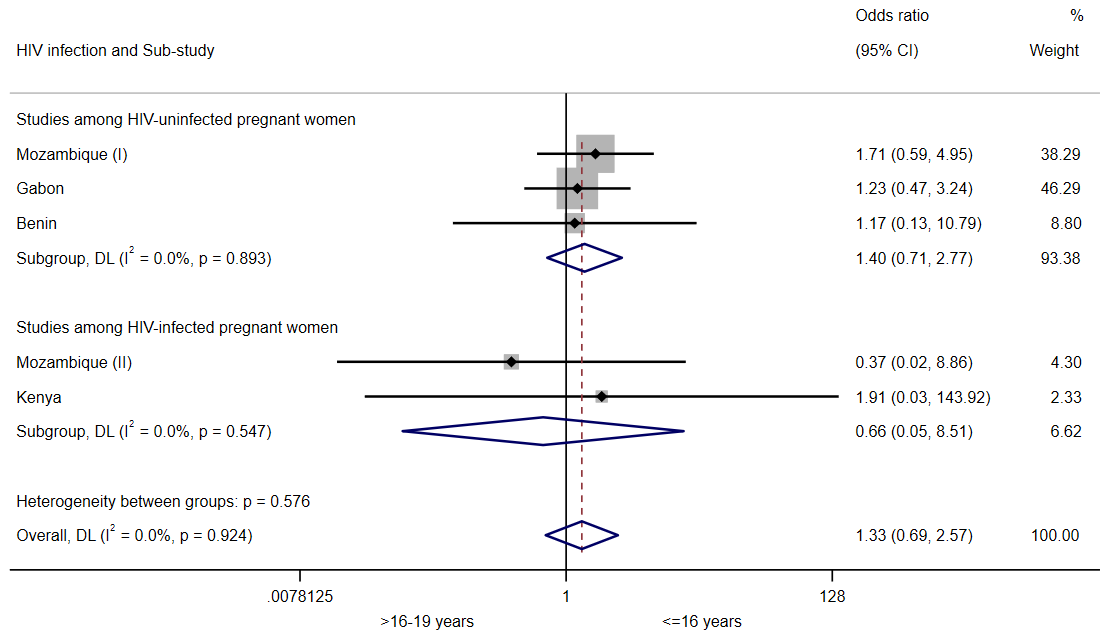
*

HIV-uninfected pregnant women, p-value=0.33

HIV-infected pregnant women, p-value=0.75

Overall, p-value=0.40

*Notes: Weights and between-subgroup heterogeneity test are from random-effects model. CI – confidence interval; DL - DerSimonian-Laird random effects model; OR – odds ratio*

#### Primary outcome: Placental malaria

- Number of included sub-studies: 6
- Total number of observations: 996

Table AA. Number of placental infection cases among adolescent girls belonging to different age groups, by sub-study

| **Sub-study** | **Overall**  n/N (%) | **≤16 years**  n/N (%) | **>16-19 years**  n/N (%) |
| --- | --- | --- | --- |
| **Mozambique (I)** | 14/404 (3.47) | 8/142 (5.63) | 6/262 (2.29) |
| **Mozambique (II)** | 3/55 (5.45) | 0/7 (0) | 3/48 (6.25) |
| **Gabon** | 25/287 (8.71) | 11/75 (14.67) | 14/212 (6.60) |
| **Tanzania (I)** | 2/139 (1.44) | 0/7 (0) | 2/132 (1.52) |
| **Benin** | 23/85 (27.06) | 1/5 (20.00) | 22/80 (27.50) |
| **Kenya** | 3/26 (11.54) | 0/2 (0) | 3/24 (12.50) |
| **Tanzania (II) *** | 0/2 (0) | - | 0/2 (0) |

** Tanzania (II), HIV-infected participants, was excluded from the analysis since no cases of placental infection were found among study participants in the site.*

Figure K. Sub-analysis among adolescent girls: placental malaria

*
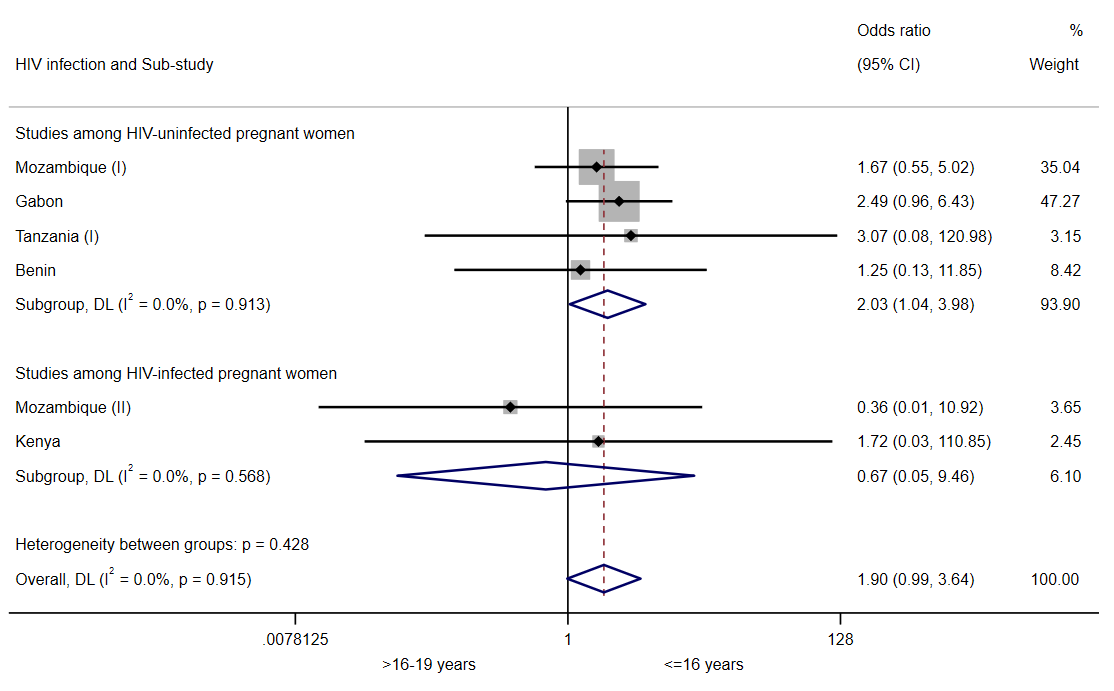
*

HIV-uninfected pregnant women, p-value=0.04

HIV-infected pregnant women, p-value=0.77

Overall, p-value=0.05

*Notes: Weights and between-subgroup heterogeneity test are from random-effects model. CI – confidence interval; DL - DerSimonian-Laird random effects model; OR – odds ratio*

#### Secondary outcome: Peripheral PCR-confirmed infection at delivery

- Number of included sub-studies: 4
- Total number of observations: 251

Table AB. Number of peripheral PCR-confirmed infections at delivery among adolescent girls belonging to different age groups, by sub-study

| **Sub-study** | **Overall**  n/N (%) | **≤16 years**  n/N (%) | **>16-19 years**  n/N (%) |
| --- | --- | --- | --- |
| **Mozambique (I)** | 7/141 (4.96) | 2/50 (4.00) | 5/91 (5.49) |
| **Gabon** | 5/37 (13.51) | 2/10 (20.00) | 3/27 (11.11) |
| **Benin** | 14/33 (42.42) | 1/4 (25.00) | 13/29 (44.83) |
| **Mozambique (II)** | 3/40 (7.5) | 0/6 (0) | 3/34 (8.82) |
| **Tanzania (I) *** | 0/5 (0) | - | 0/5 (0) |
| **Tanzania (II) *** | - | - | - |
| **Kenya *** | 0/15 (0) | 0/2 (0) | 0/13 (0) |

** Tanzania (I), HIV-uninfected participants, was excluded from the analysis since no cases of PCR-confirmed peripheral infection at delivery were found among study participants in the site. Tanzania (II), HIV-infected participants, was excluded since no PCR test were performed among the study participants of the site. Kenya was as well excluded from the analysis since no cases of PCR-confirmed parasitaemia at delivery were reported among adolescent girls in the site.*

*PCR – polymerase chain reaction*

Figure L. Sub-analysis among adolescent girls: peripheral PCR-confirmed infection at delivery

*
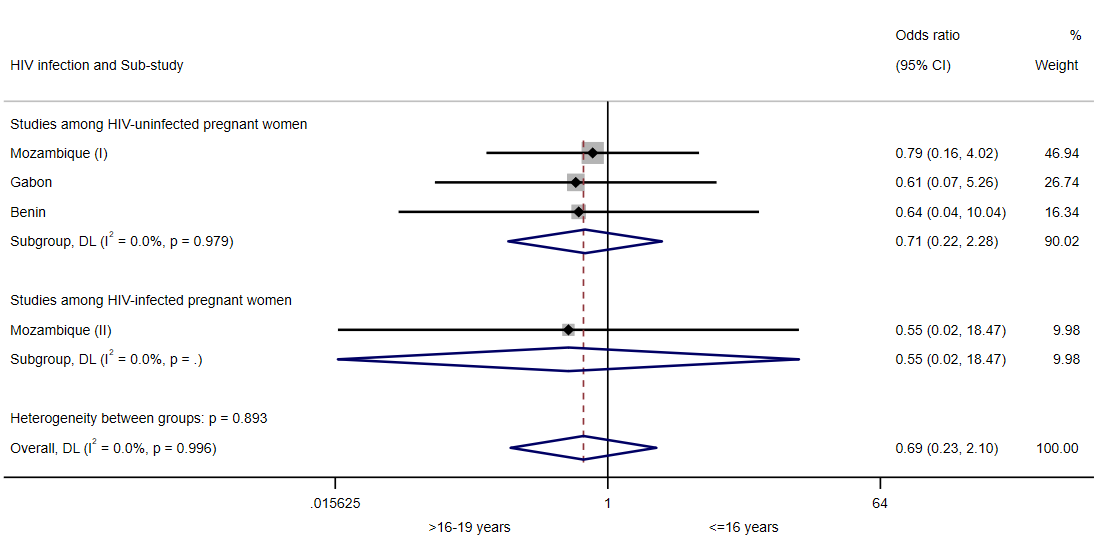
*

HIV-uninfected pregnant women, p-value=0.56

HIV-infected pregnant women, p-value=0.74

Overall, p-value=0.51

*Notes: Weights and between-subgroup heterogeneity test are from random-effects model. CI – confidence interval; DL - DerSimonian-Laird random effects model; OR – odds ratio; PCR – polymerase chain reaction*
